# Structure‐Guided Optimization of PDE9A Inhibitors Tackling Neuroinflammation: A Combined Computational and In Vitro Study

**DOI:** 10.1002/cmdc.70461

**Published:** 2026-08-31

**Authors:** Giovanni Ribaudo, Elisa Landucci, Margrate Anyanwu, Costanza Mazzantini, Matteo Giannangeli, Stefano Rosa, Francesca Mainolfi, Maura Calvani, Maurizio Memo, Domenico E. Pellegrini‐Giampietro, Alessandra Gianoncelli

**Affiliations:** ^1^ Department of Molecular and Translational Medicine Università di Brescia Brescia Italy; ^2^ Department of Health Sciences Section of Clinical Pharmacology and Oncology University of Florence Florence Italy; ^3^ Department of Pediatric Hematology‐Oncology Meyer Children’s Hospital IRCCS Florence Italy

**Keywords:** flavonoids, molecular dynamics, neurodegeneration, neuroinflammation, PDE9A

## Abstract

Oxidative stress and inflammation are key players in central nervous system (CNS) diseases and in neurodegeneration. In this field, the search for novel targets and therapeutic tools is wide open. In this study, the identification of potential inhibitors of phosphodiesterase 9 (PDE9), a target of growing interest in CNS diseases, was done by using a rational virtual screening approach that employed ligand‐ and structure‐based methodologies. Furthermore, a novel method to evaluate PDE9A activity based on high‐performance liquid chromatography (HPLC) was developed to address the need for a time‐ and cost‐effective assay to evaluate inhibitors. One of the candidates identified by virtual screening demonstrated potent enzymatic inhibition, and subsequent in vitro tests proved its significant PDE9A‐dependent anti‐neuroinflammatory effects as it acted on pro‐inflammatory mediators such as COX‐2, IL‐1β, TNFα, and IL‐6. The results of this multidisciplinary study underscore the potential of developing PDE9A inhibitors to modulate cyclic guanosine monophosphate (cGMP) signaling pathways implicated in neuroinflammation and also potentially in synaptic plasticity and cognitive functions, paving the way for novel PDE9A‐targeting inhibitors addressing neurodegenerative diseases.

## Graphical abstract

Computational studies allowed the identification of a PDE9A inhibitor. A novel HPLC‐based assay was developed and optimized to measure enzyme function to assess PDE9A inhibition. Furthermore, in vitro tests supported the activity of the compound against neuroinflammation.

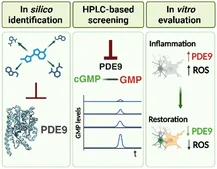

## 1 Introduction

Neuroinflammation plays a key role in the progression of neurodegenerative conditions, driving neuronal damage and cognitive decline [1, 2, 3]. In this context, dementia is characterized by a group of symptoms that gradually reduce the ability to perform daily tasks and eventually result in a loss of personal autonomy [4]. Indeed, it is more considered as a syndrome rather than as a disease itself, and it represents the seventh leading cause of death around the world [4, 5]. As life expectancy rises, according to the World Health Organization, dementia deaths are expected to increase from 55 million in 2019–139 million by 2050 [6].

Phosphodiesterases (PDEs) are encoded by 11 gene families (PDE1 to PDE11), including more than 100 different proteins or isoenzymes [7]. As detailed below, PDE9 enzymes are expressed in various regions of the human brain, and in the past few years, several inhibitors have shown results in AD models. For example, cilostazol and milrinone (targeting PDE3) or sildenafil and tadalafil (targeting PDE5) were reported to promote beneficial effects on cognition and memory recovery [8]. More specifically, the in vitro results demonstrated that milrinone could inhibit the secretion of interleukin‐1 beta (IL‐1β), IL‐6, and tumor necrosis factor‐alpha (TNF‐α) by regulating the NLRP3 inflammasome and TLR4/MyD88/NF‐κB signaling pathway [9].

In general, PDEs act by hydrolyzing their substrates, cyclic adenosine monophosphate (cAMP) and/or cyclic guanosine monophosphate (cGMP), into their linear analogs. Indeed, in dementia, altered cAMP/cGMP levels are associated with cognitive impairment in neurodegenerative disorders [10]. Furthermore, Nabavi et al. demonstrated that dysfunctional PDE activity can disrupt signal transduction pathways, leading to abnormal levels of cAMP and cGMP in brain regions associated with memory. This imbalance contributes to increased amyloid beta (Aβ) production and impairs memory formation [11]. Hence, the level of cAMP in the cytoplasm is critical for modulating the events of neurotransmitter vesicles, as well as synaptic transmission and neuronal plasticity [12]. On the other hand, cGMP is peculiarly connected to the NO/cGMP/protein kinase G (PKG) pathway, which is responsible for the regulation of neurotransmitter release and long‐term (LT) changes of synaptic activity in different brain regions by activating the effector PKG. This protein kinase triggers the transcription of cAMP response element‐binding protein (CREB), thus promoting neurotransmission, synaptic plasticity, and memory formation [13]. In fact, the activation of CREB leads to the transcription of specific genes coding for growth factors such as brain‐derived neurotrophic factor (BDNF), which plays an essential role in synaptogenesis and proliferation, survival, and differentiation of new neurons [14, 15].

Several studies have shown that inhibiting PDE isoforms in specific brain regions activates cGMP‐PKG signaling and leads to the phosphorylation of CREB, enhancing impaired neuroplasticity and memory deficits in AD [7, 16]. PDE9 is a cGMP‐specific PDE, discovered in 1998, with the highest affinity for cGMP (*K*_m_ of 70–170 nM) and selectivity over cAMP (*K*_m_ of 230 mM) [17]. The PDE9A gene is located on chromosome 21q23.3 and contains over 20 exons that encode 21 N‐terminal mRNA variants [18]. Based on its localization in cortical and hippocampal tissues and, at the cellular level, in glial cells, in neuronal cell bodies, and in primary dendrites, which indicates postsynaptic placement, PDE9 has become a promising therapeutic target for modulating cyclic nucleotide signaling pathways involved in neuronal plasticity and inflammation [19, 20, 21, 22, 23].

The study aims to identify PDE9 inhibitors that modulate neuroinflammatory processes and to elucidate their mechanisms of action in vitro. To this end, we employed a virtual screening strategy based on a rational substructure search starting from DB987, a compound that we recently reported [20]. The screening workflow combined ligand‐ and structure‐based approaches, leading to the identification of two promising candidates. To facilitate rapid evaluation, we developed an efficient, high‐performance liquid chromatography (HPLC)‐based enzymatic assay to assess the PDE9 inhibitory activity of discovered compounds. Finally, comprehensive in vitro characterization enabled validation of the biological activity and PDE9‐dependent anti‐neuroinflammatory effects of the investigated compounds.

## 2 Results and Discussion

### 2.1 Identification of PDE9 Binders by Ligand‐Based Virtual Screening

**FIGURE 1:**
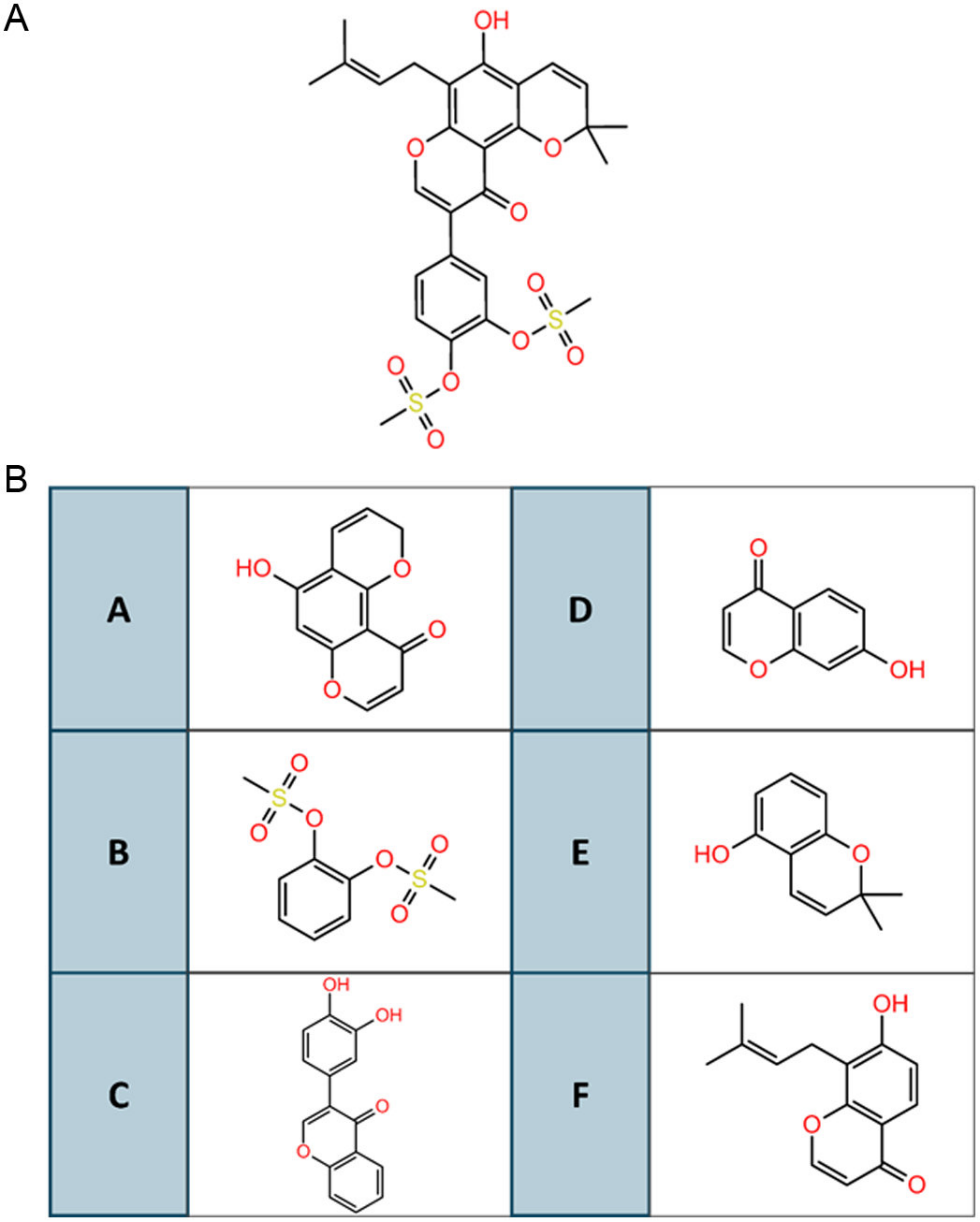
Structure of DB987 used as a reference for query generation (A) and overview of structural queries (B) obtained from DB987 used for in‐house database research.

Building on the findings from our recent study, in which DB987, a modified derivative of pomiferin extracted from *Maclura pomifera*, was identified as a potent PDE9 inhibitor (IC_50_ = 5.35 nM), we performed a substructural search analysis on our in‐house database using this compound as a reference molecule (Figure 1A) [20, 24].

Our objective was to retrieve effective PDE9 inhibitors capable of mitigating neuronal inflammation, a hallmark of numerous neurodegenerative disorders such as AD and Parkinson’s disease, in which PDE9A expression is shown to be increased [25, 26]. In addition, this approach aimed to provide further insight into the pharmacophoric features involved in the activity of structurally related PDE9 inhibitors.

The rationale for selecting PDE9 inhibitors includes a substructure search, molecular docking, filtering best‐docked compounds that interact with the PDE9 binding site via key residues, and stability assessment via molecular dynamics (MD) simulation.

More specifically, the criteria used to define the structural queries derived from DB987 included the presence of at least eight carbon atoms and two heteroatoms. These criteria allowed the generation of six queries (A–F) based on the chemical structure of DB987 (Figure 1B). The queries were then used to screen the in‐house database comprising 672 compounds, yielding 27 molecules that matched the search criteria (Figure S1). Thus, this filtering step reduced the number of candidates to be carried to the molecular docking analysis.

### 2.2 Structure of PDE9A and Molecular Docking

PDEs are encoded by 11 gene families (PDE1 to PDE11), where each includes 1–4 different genes (21 in mammals) that result in more than 100 different isoforms. PDEs generally exist as dimers in which each monomer shares three structural features: an N‐terminal regulatory domain that characterizes the presence of variants in each family; a catalytic domain composed of almost 340 conserved residues; and a C‐terminal domain on which post‐translational modifications (PTMs) like phosphorylation or prenylation can occur [27]. Due to these structural differences, PDEs have different biochemical properties, which can also be related to their tissue, cellular, and subcellular distribution. From the functional point of view, PDEs can be divided into three families: cAMP‐selective, cGMP‐selective, and dual‐selective PDEs, in which some of those stand out for their high selectivity for these second messengers [28]. PDE9 isoforms are differently expressed across subcellular domains in the cell across different tissues [27]. Due to high PDE9A expression at the level of the CNS and its involvement in several pathological pathways, PDE9A inhibitors started to gain some relevance.

From a structural point of view, PDE9A exists as a dimer in which each monomer is composed of a coiled N‐terminal region and a conserved catalytic domain at the C‐terminal region. PDE9A binding site is composed of three sub‐regions: the purine‐selective glutamine and hydrophobic clamp (Q) pocket, the metal (M) pocket, and the hydrophobic (H) pocket. The first contains residues Gln453 and Phe456, which form key hydrogen bonds and π–π stacking interactions with the purine core of cGMP. Then, Glu406 has the role of stabilizing the side chain of Gln453, which, when targeted, contributes to selectivity in the rational design of inhibitors. Then, the M pocket contains a network of water molecules, five histidines (His252, His296, His325, His292, and His256), and two aspartate residues (Asp293 and Asp402) that play a role in mediating catalysis. Here, Mg^2+^ and Zn^2+^ are present and allow for the establishment of an octahedral coordination geometry to further stabilize this region. Lastly, the H pocket is a small, deep region where hydrophobic residues such as Val417, Leu420, Leu421, Phe441, Ala452, and Val447 are present. Notably, Tyr424 is present in the H pocket of both PDE8 and PDE9 but not in other PDEs, making it a key residue for achieving selectivity [29].

**FIGURE 2:**
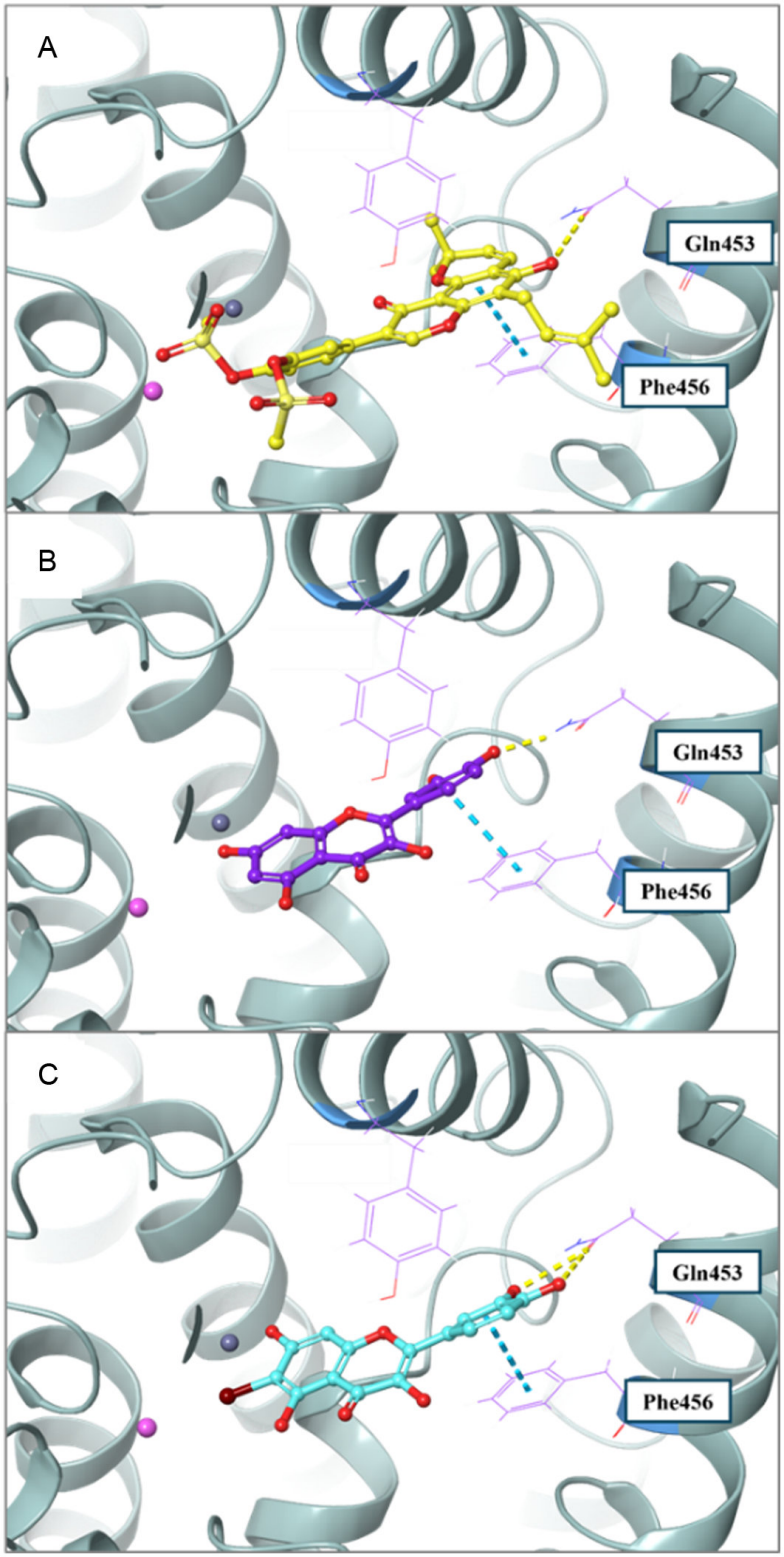
Docking poses of DB987 (A) [20], BS586 (B), and BS560 (C), showing key interactions with Gln453 (highlighted in yellow for hydrogen bonding) and Phe456 (highlighted in light blue for π–π stacking).

In our virtual screening workflow, the first step was to dock the 27 compounds resulting from the substructure search (reported in Figure S1) to the PDE9A enzyme and its cognate ligand, PF‐04447943, whose structure was retrieved from the Protein Data Bank (PDB ID: 4E90). Top binders resulting from molecular docking (BS590, BS563, BS586, BS579, BS570, BS560, BS570, BS577, and BS571) were evaluated in terms of docking score, and the nature of their interaction was established with the target. Specifically, interactions with Gln453 and Phe456 were used as selection criteria, allowing the identification of compounds BS586 and BS560, quercetin, and a 6‐bromoquercetin, respectively. In this context, PF‐04447943 formed strong hydrogen bonds and hydrophobic interactions with key residues, such as Gln453 and Phe456, supporting the relevance of these residues [20, 30]. The reference compound DB987 (−8.782 kcal/mol) established a hydrogen bond with His296, where the Hε2 atom of His296 served as the donor and the carbonyl oxygen (O_16_) of the methanesulfonate group, which acted as the acceptor, thereby resulting in a strong hydrogen bond with a length of 1.86 Å (Figure 2A). Similarly, the hydrogen atoms Hδ21 of Asn300 and the H atom of Asn301 functioned as donors in hydrogen bonds with the carbonyl oxygens O_38_ and O_37_ of the methanesulfonate moieties, at distances of 2.68 and 2.44 Å, respectively. Two additional key interactions contributed to the stabilization of DB987, reinforcing its potential as a promising starting compound, where the hydroxyl oxygen (O_19_) of DB987 served as a hydrogen bond acceptor with the Oε1 atom of Gln453, exhibiting a bond length of 1.77 Å (thus indicating a strong interaction), and another relevant interaction included a π–π stacking interaction between aromatic ring A of the isoflavone scaffold and the aromatic side chain of Phe456, with a ring‐to‐ring distance of 3.59 Å (Figure S2A). Compound BS586 (−9.088 kcal/mol) exhibited interactions between its B ring and both key residues (Figure 2B). First, a hydrogen bond in which oxygen in the hydroxyl group (O_20_) served as acceptor for the interaction with the Hε22 in Gln453 (1.95 Å). Additionally, the B ring established a π–π stacking interaction with the phenyl ring in Phe456 (4.17 Å) and a hydrogen bond between O_22_ in the hydroxyl moiety of the B ring and the carbonyl moiety in the Ile403 backbone (1.88 Å). Then, the A ring allowed for the formation of a π–π stacking interaction with the imidazole ring in His252 side chain (5.08 Å), and the C ring allowed for salt bridge and metal coordination formation between atom O_19_ and Zn^2+^ and Mg^2+^ present in the M pocket (Figure S2B). Then, among the top compounds evaluated through molecular docking, BS560 (−8.360 kcal/mol), stood out for its interactions established with key residues Gln453 and Phe456 (Figure 2C). Two hydrogen bonds were formed between O_21_ and O_23_ in the B ring and acted as donors to the Oε1 atom of the Gln453 side chain, with bond lengths of 1.96 and 2.08 Å, respectively. The same ring established a π–π stacking interaction with the Phe456 side chain (4.08 Å). Further, ring A contributed in BS560 through a hydrogen bond between backbone Thr363 and O_7_ (2.67 Å) and by a π–π stacking interaction with the imidazole in His252 (5.05 Å) (Figure S2C). Eventually, a metal coordination (2.09 Å) and a salt bridge (2.09 Å) were established between O_7_ and the Zn^2+^ atom. Compounds BS590 (−9.550 kcal/mol), BS563 (−9.177 kcal/mol), and BS570 (−8.607 kcal/mol) were discarded since they did not establish relevant interactions with both PDE9A residues.

### 2.3 MD Simulation

To evaluate whether the interactions established during molecular docking allowed the formation of a transient or stable complex, classical MD runs were performed, using the docked poses of BS586/PDE9A and BS560/PDE9A as starting points for 500 ns simulations.

**FIGURE 3:**
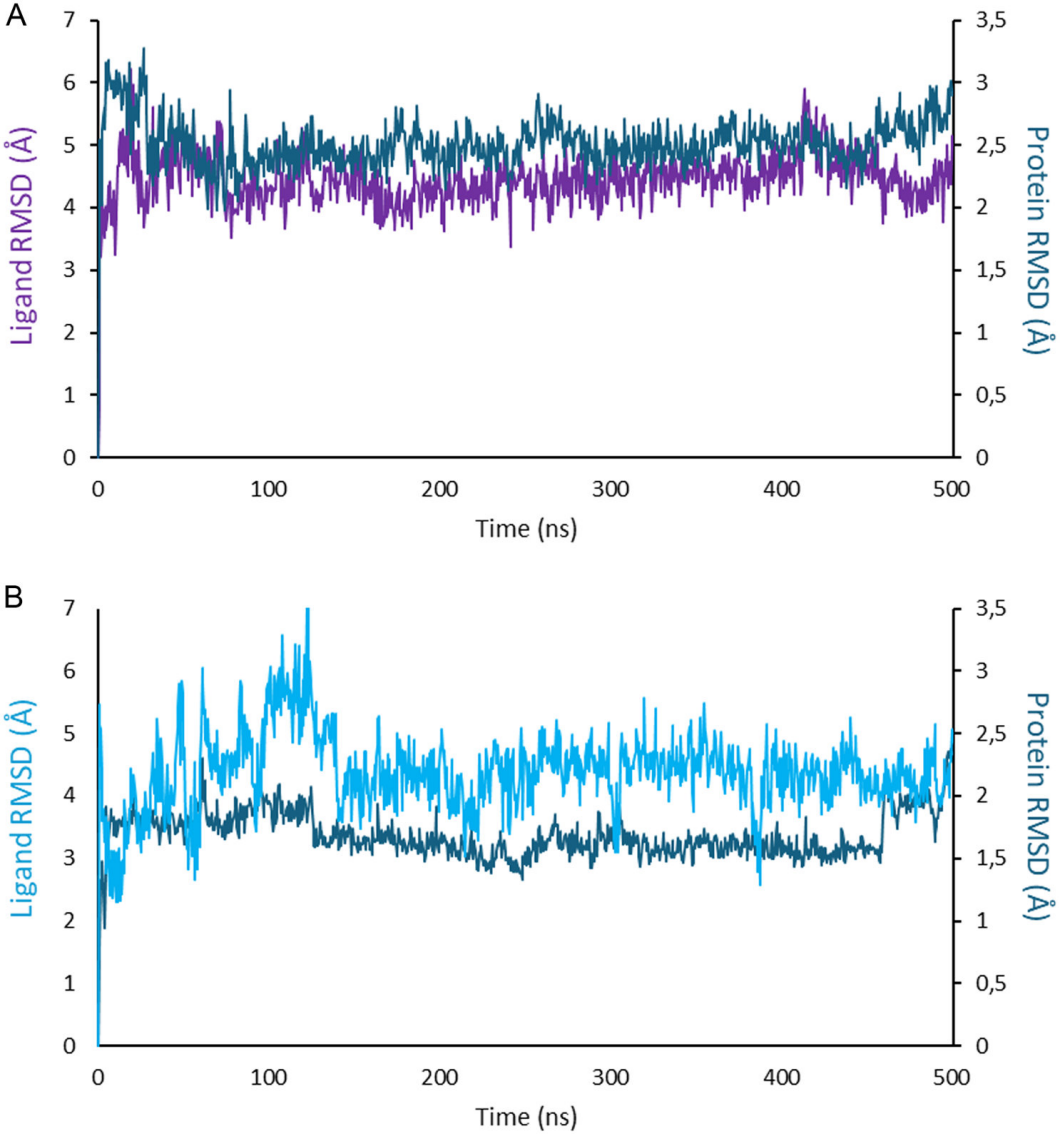
RMSD profiles for PDE9A (dark green) in complex with BS586 (A) and BS560 (B). RMSD profiles of ligands are depicted in purple (A) and cyan (B). The 500 ns simulation shows the formation of a stable complex with no significant conformational changes in the protein structure.

For BS586/PDE9A, the trajectories showed marked stability in both the protein and ligand systems, with only minor fluctuations (Figure 3A). By examining interactions that lasted for 30% of the simulation time, the rings A and C in the ligand were well inserted into the M and H pockets, where they interacted through π‐cation and π–π stacking interactions with ring *A* and Lys399. Then, the O_19_ established interactions with Zn^2+^, further involving metal coordination with Asp293, His256, Asp402, and His292, supporting a good insertion in the M pocket. Further, the interaction between Hε22 in Gln453 and O_5_ in BS586 supported the stability of the complex. When checking the B ring in BS586, this showed a highly stable and rigid structure given by a set of hydrogen bonds partially mediated by water molecules between Glu406, Ala413, Asn405, and O_20_, and between Ile403 and O_22_ (Figure S3A). The set of these interactions effectively minimized fluctuations in BS586, keeping it stably within the binding pocket throughout the simulation, as demonstrated by Raoot‐mean‐square fluctuation (RMSF) trajectories reported in Figure S4A.

Then, the BS560/PDE9A complex was evaluated. Unlike BS586, the root‐mean‐square deviation (RMSD) plot of the complex showed considerable oscillations during the first 150 ns, which attenuated over the course of the simulation. Further, RMSD analysis of the final frames showed greater protein oscillation at 460 ns, attributed to the C‐terminal helix (residues 481–503) (Figure 3B). By examining the interactions present in at least 30% of the simulation, a metal coordination center was identified between Zn^2+^ in the protein, Br, and the O_7_ atoms in ring A, which supported the interactions with Asp293, His256, His292, and Asp402. Further, O_5_ in ring A established a hydrogen bond with Thr363. Then, ring C interacted with Tyr424 aromatic ring via a π–π stacking interaction; O_11_ formed a water‐mediated hydrogen bond with Asp364, and a hydrogen bond with Met365 was established for 34% of the simulation. Another relevant interaction between Gln453 and the O_23_ atom in ring B was detected, suggesting a stabilizing effect of the ring in the complex (Figure S3B). Different from BS586, BS560 showed a spike associated with the B ring in the RMSF profile attributable to the rotation of the single C_15_─C_16_ bond (Figure S4B). An analysis of key residues during simulations of both BS586 and BS560 in complex with PDE9A reveals distinct binding modes for the two compounds. In the case of the BS586/PDE9A complex, initial hydrogen bonds were established between Tyr424 and the C ring of the ligand, while Gln453 and Phe456 remained uninvolved until, over time, Tyr424 gradually reoriented and moved away, reducing the possibility of further interactions. Although Phe456 stayed close to the ligand, its orientation was not ideal for maintaining the binding throughout the simulation. In contrast, Gln453 maintained a stable and direct interaction with this ring. As a result, while the complex remained structurally stable, its configuration offered limited support for a potential inhibitory effect on PDE9A (Figure S5). Conversely, the orientation of the bromine atom toward the M pocket strongly favors halogen bond formation, aiding in the stabilization of the quercetin scaffold. In the BS560/PDE9A complex simulation, the Tyr424 residue (critical for PDE9A inhibition and selectivity) positions its side chain parallel to the ligand’s C ring, promoting a π–π stacking interaction; likewise, the B ring and the side chain of Phe456 (Figure S6) [27]. These stacking interactions help stabilize the binding site geometry and correctly orient Gln453, enabling the formation of stable hydrogen bonds and highlighting BS560 as the best candidate in the set. Finally, in silico  Absorption, distribution, metabolism, and excretion (ADME) prediction was performed, resulting in no violation of Lipinski’s rule of five. Furthermore, no passive BBB crossing was predicted; however, alternative active transport cannot be ruled out for the compound (Figure S11).

### 2.4 Development of Enzymatic Screening Method and Evaluation of the Compounds

To evaluate in vitro the inhibitory activity of compounds identified by virtual screening, a label‐free, HPLC‐based enzymatic assay was developed, optimized, and applied in this study. This approach is based on monitoring the decrease in cGMP concentration upon incubation with PDE9A and ligands, and it was inspired by other PDE assays involving various techniques, including nuclear magnetic resonance (NMR) spectroscopy and liquid chromatography‐mass spectrometry (LC/MS), previously reported in the literature, and was designed to be cost‐effective, rapid, and easy to perform in‐house [20, 31, 32, 33]. Compared to other methods, this approach provides several practical advantages, such as reduced instrumental and operational costs, and allows direct measurement of substrate and product without requiring radiolabels, fluorescent probes, or specialized detection systems. These features make the assay ideal for routine in‐house screening and validation studies. At the same time, the method has some limitations, including lower throughput than plate‐based fluorescence assays, longer analysis times due to the required chromatographic separation, and the need for a chromophore in the compounds under investigation with the current experimental setup. Nevertheless, it provides a robust and accessible alternative for assessing PDE9A inhibition.

**FIGURE 4:**
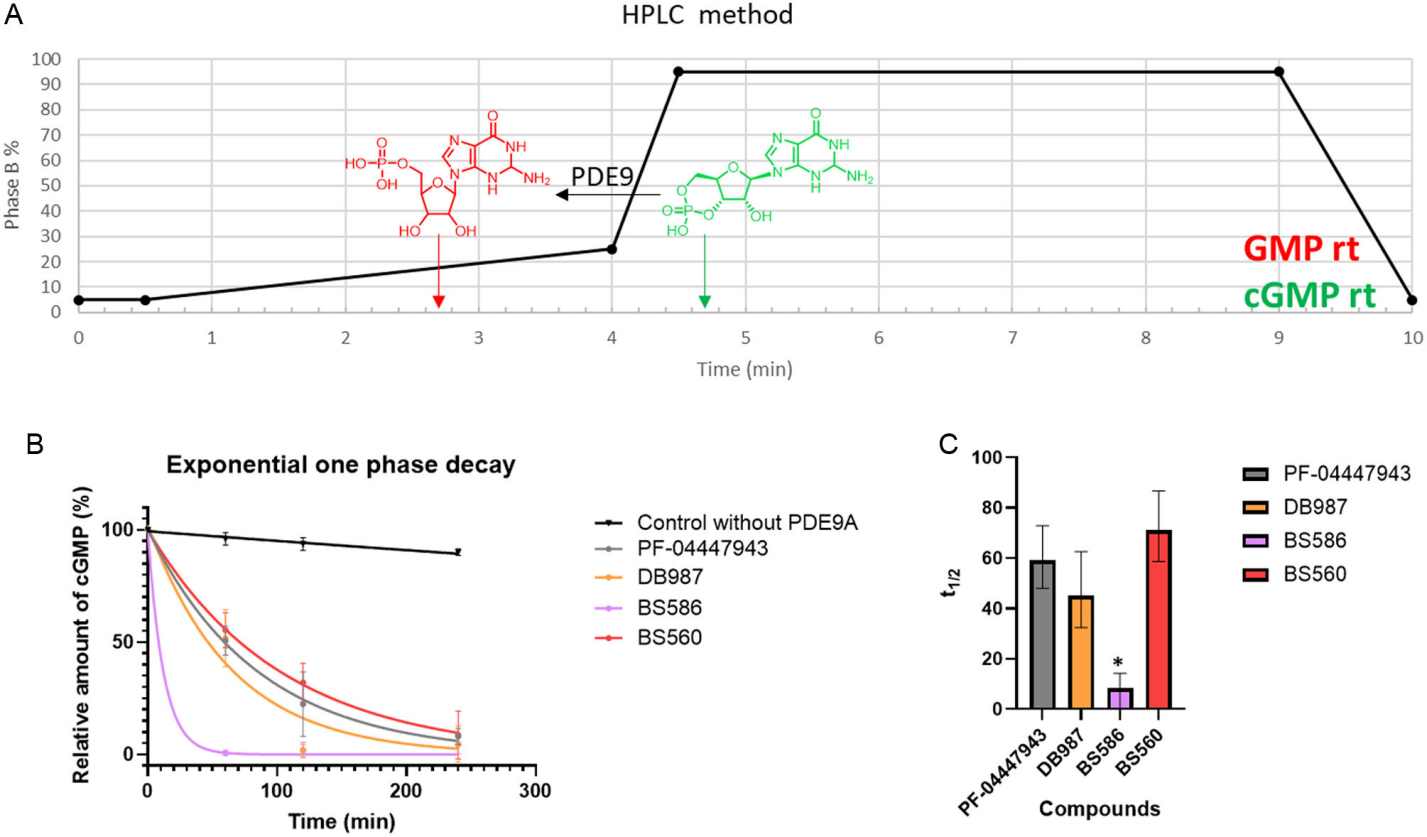
(A) HPLC gradient program (% phase B, acetonitrile, vs. time) used for monitoring the enzymatic reaction, indicating the retention times of the substrate cGMP and product (GMP). (B) Exponential one‐phase decay of cGMP relative concentration over time in the presence of different inhibitors. Curves represent the mean of three independent experiments ± SD. Solid lines correspond to nonlinear regression fits to a one‐phase exponential decay model. The negative control experiments (cGMP and PDE9A without ligands) are not shown since they are out of range, as at the first time point, there was no detectable cGMP (Figure S8). (C) Results of the inhibition assay with data expressed as t_1/2_, with error bars indicating 95% confidence intervals. PF‐04447943 was employed as a positive control. Furthermore, tadalafil, which is selective for PDE5 and has a 20 k‐fold selectivity over PDE9 [35], was employed as a negative control (Figure S10). Bars represent the mean ± SD of three experiments. *p < 0.05 versus PF‐04447943 (one‐way ANOVA followed by Dunnett’s test).

An HPLC method able to clearly resolve cGMP and GMP in a reasonable time had to be developed (the detailed method is reported in the Experimental section). Different conditions were tested; in particular, the stability of cGMP in the buffer under experimental conditions was verified, and no GMP was detected after 4 h (Figure S7). The time intervals, time span, and concentration of the substrate and PDE9A were chosen based on the enzymatic activity provided by the manufacturer (≥97 pmol/min/μg); in fact, by considering this data, in our experimental setup, the enzyme should have been able to convert all cGMP in about 4 h. However, when this experiment was repeated in the presence of PDE9A, cGMP was completely converted to GMP within 1 h (t1). Indeed, a *V*_max_ of 960–4900 pmol/min/μg for PDE9A is reported in the literature, much higher than the one provided by the manufacturer [34]. However, it was decided to continue with this setup, since, as detailed in the following, it allowed us to work in a proper range to assess whether a compound had inhibitory activity or not. Consequently, PF‐04447943, a known PDE9A inhibitor, was tested as a positive control, successfully inhibiting the enzyme compared to the experiment without ligands. Then, three compounds from our in‐house database, DB987 (which was already reported as a promising PDE9A inhibitor) [20], BS586, and BS560, were assayed, showing different PDE9A‐inhibiting activity (Figure 4A). The results were expressed as *t*_1/2_ (more details are reported in the Experimental section), the time required for the enzyme to convert 50% of cGMP into GMP in this experimental setup. BS586 showed the worst *t*_1/2_ (8.4 min), while DB987 and BS560 showed *t*_1/2_ results in the same range of PF‐04447943 (respectively 45.14, 71.12, and 59.04 min) as shown in Figure 4B. The results of this experiment, which allowed us to assess computational binding data, testify that BS560 inhibits the enzyme in vitro in a manner comparable to that of positive controls (Figure 4C). Representative chromatograms, including negative and positive controls are reported in Figures S7–S10.

### 2.5 Assessment of PDE9A Expression in Rat Mixed Glial Cells

**FIGURE 5:**
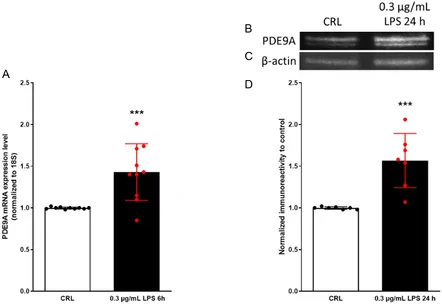
PDE9A increased in rat mixed glial cells exposed to LPS. The mRNA levels of PDE9A were increased in glial cells treated with 0.3 µg/mL LPS for 6 h (A). Illustrative blots using antibodies directed against PDE9A (B) and β‐actin (C). Quantitative analysis of immunoreactive bands shows a significant increase in protein levels of PDE9A in glial cells treated with LPS for 24 h (D). Bars represent the mean ± SD from ten (A) and seven (D) independent experiments from different glial cell preparations, and each dot is the pool of two wells. ***p < 0.001 versus CRL. (Unpaired t‐test in (A and D).

In our previous study, we observed that PDE9A mRNA is elevated in organotypic hippocampal slices exposed to kainic acid [20]. Moreover, PDE9A mRNA is elevated in the aged human hippocampus with dementia when there is a history of traumatic brain injury [36]. In this study, the expression of PDE9A was detected in rat mixed glial cells in the control condition and after exposure to bacterial lipopolysaccharides (LPS). LPS treatment is a widely accepted and highly robust model to study neuroinflammation, a core pathological hallmark shared by Alzheimer’s and Parkinson’s diseases. Clinical data have directly identified bacterial LPS within amyloid plaques in postmortem human brains, confirming its pathophysiological relevance. By activating the TLR4 pathway, this model effectively reproduces the reactive gliosis, oxidative stress, and pro‐inflammatory cytokine profile (TNF‐α, IL‐1β, IL‐6) that drive neuronal death in patients [37]. Therefore, despite its limitations, this model remains an invaluable tool for isolating neuroinflammatory mechanisms and screening potential therapeutic compounds. The gene and protein expression of PDE9A was evaluated by quantitative real‐time polymerase chain reaction (RT‐qPCR) and western blot, as shown in Figure 5, where we observed a significant increase in the level of gene and protein PDE9A in cells exposed to 0.3 µg/mL for 6 and 24 h, respectively (Figure 5A–D). These studies have therefore allowed us to identify the involvement of PDE9 in neuroinflammation.

### 2.6 Effects of PDE9A Inhibitors on Mixed Cortical Glial Cells Viability

**FIGURE 6:**
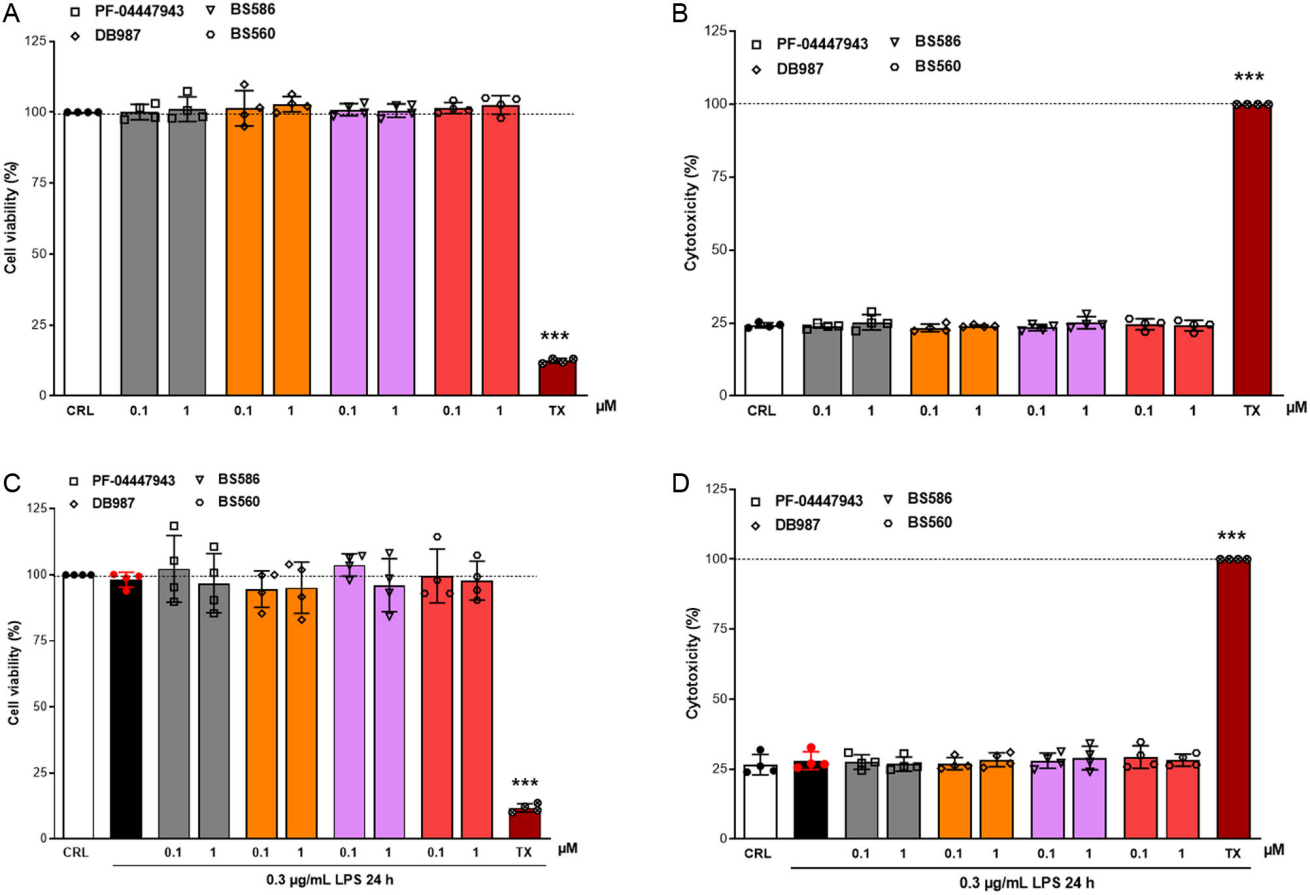
Rat mixed glial cells viability evaluated by MTT assay (A and C) and cytotoxicity by LDH assay (B,D). Glial cells were exposed for 24 h to PF‐04447943, DB987, BS586, and BS560 (0.1–1 µM) (A,B) or to LPS 0.3 µg/mL in the presence of PDE9A inhibitors (C,D). Data are expressed as a percentage of control (CRL) and Triton X‐100 (TX), which represent, respectively, the maximum cell viability and cell cytotoxicity. Values represent the mean ± SD of at least four experiments performed in duplicate. ***p < 0.001 versus CRL (one‐way ANOVA followed by Dunnett’s test).

To evaluate the safety and tolerability of PDE9A inhibitors, cytotoxicity and cell viability assays were performed in rat mixed glial cells. Glial cells, approximately 70%–80% confluent, were incubated for 24 h with PF‐04447943, DB987, BS586, and BS560 at concentrations of 0.1 and 1 µM in the presence or absence of 0.3 µg/mL LPS. Triton X‐100 was considered the negative control. Cell viability and cell damage were assessed by MTT assays and by measuring the release of lactate dehydrogenase (LDH), respectively (Figure 6). In MTT‐based cell viability assays, no significant differences were observed between cells treated with PDE9A inhibitors at both concentrations tested. Also, LPS did not alter the cell viability when present in the incubation medium alone or in the presence of PDE9A inhibitors, compared to the control (CRL) cells (Figure 6A,C). Furthermore, no significant differences in LDH levels, an indicator of cell death and cytotoxicity, were observed in rat mixed glial cells treated with PDE9A inhibitors alone or in the presence of LPS for 24 h, compared to the vehicle (Figure 6B,D). No significant toxicity was observed after treatment with the compounds.

**FIGURE 7:**
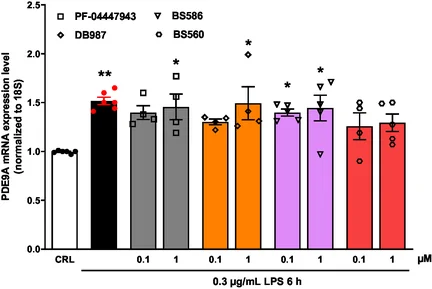
The mRNA levels of PDE9A in glial cells exposed to LPS and PDE9A inhibitors. PF‐04447943, DB987, BS586, and BS560 did not alter the increased levels of the PDE9A gene induced by 0.3 µg/mL of LPS for 6 h. Bars represent the mean ± SD from at least four independent experiments from different glial cell preparations, and each dot is the pool of two wells. *p < 0.05 and **p < 0.01 versus CRL (ANOVA + Tukey’s w test).

To better investigate the effects of PDE9A inhibitors on the increased mRNA levels of PDE9A induced by 0.3 µg/mL LPS for 6 h, we analyzed by RT‐qPCR the expression levels of PDE9A following the incubation with LPS in the presence of PDE9A inhibitors at concentrations of 0.1 and 1 µM in rat mixed glial cells. In a new experimental set, we confirmed that LPS treatment for 6 h induced a significant increase in PDE9A mRNA expression levels and showed that the PDE9A inhibitors did not significantly alter the increased levels induced by LPS (Figure 7).

### 2.7 Anti‐Inflammatory Effects of PDE9A Inhibitors on LPS‐Stimulated Glial Cells

**FIGURE 8:**
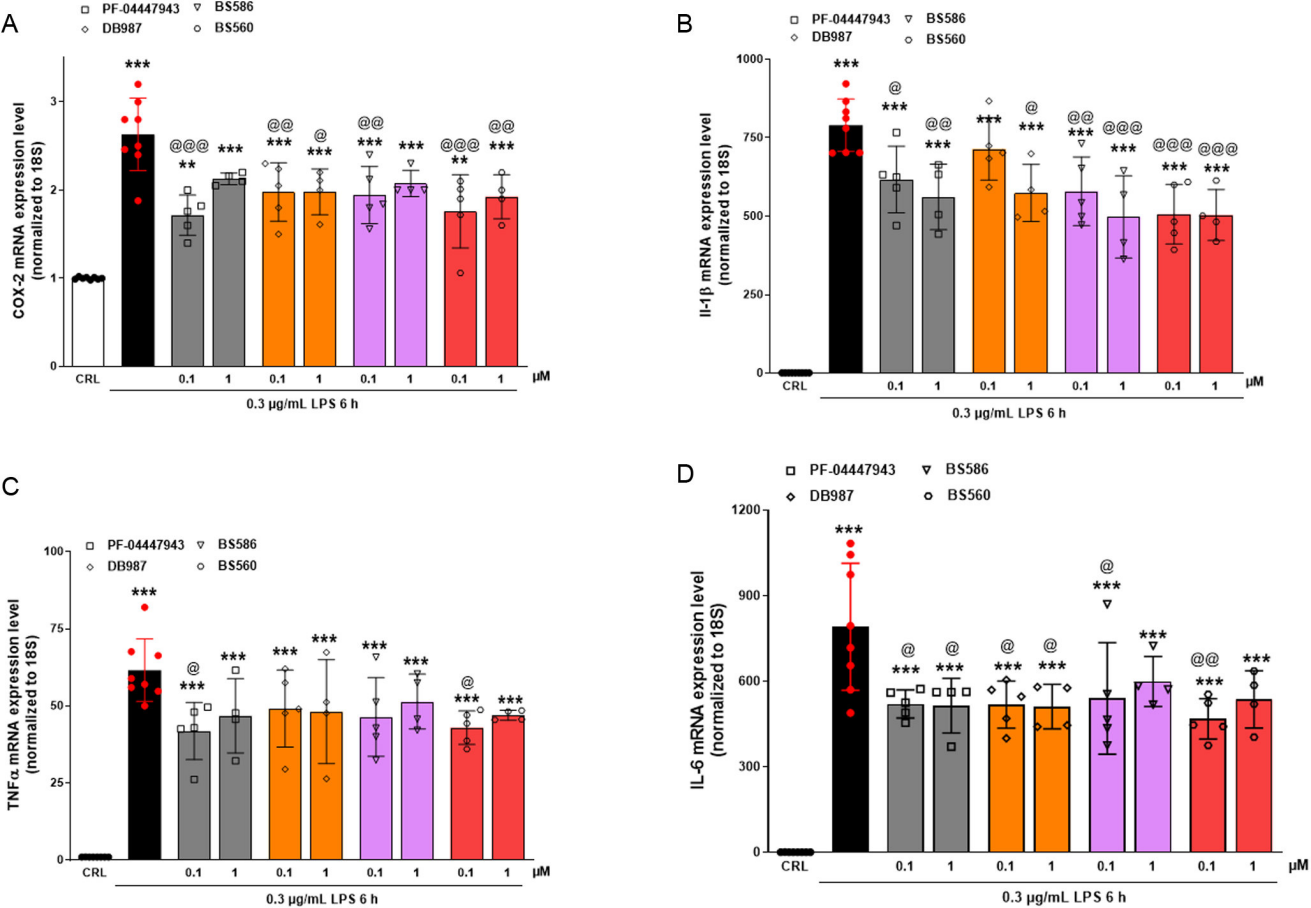
The mRNA levels of COX‐2, IL‐1β, TNFα, and IL‐6 in glial cells exposed to LPS. PDE9A inhibitors significantly reduced the increased levels of proinflammatory gene induced by 0.3 µg/mL of LPS for 6 h. Bars represent the mean ± SD from at least four independent experiments from different glial cell preparations, and each dot is the pool of two wells. **p < 0.01 and ***p < 0.001 versus CRL; @ *p* < 0.05, @@ *p* < 0.01, and @@@ *p* < 0.001 versus LPS (ANOVA + Tukey’s w test).

In our previous studies, we observed that PDE9A inhibitors reduced the damage induced by kainic acid in the CA3 regions of organotypic hippocampal slices, as evaluated by propidium iodide (PI) [20]. To establish the involvement of PDE9A in neuroinflammatory disorders, in this study, we used LPS‐treated mixed glial cells as an in vitro model, which was used specifically to study neuroinflammation [38, 39]. LPS exposure induces the activation of the glial cells that mimics the reactive state observed in AD brains. The activation of the cells induces the robust production and release of pro‐inflammatory mediators, such as IL‐1β, IL‐6, and TNF‐α [6, 40, 41]. Based on these considerations, we analyzed the anti‐inflammatory effect of PDE9A inhibitors under LPS exposure. Figure 8 shows that in mixed glial cells exposed to 0.3 µg/mL LPS for 6 h, a significant increase in the mRNA expression of the pro‐inflammatory mediators such as COX‐2, IL‐1β, TNFα, and IL‐6 compared to control, as previously observed in in vitro [42] and in vivo studies [43]. However, when present in the incubation medium, PF‐04447943, DB987, BS586, and BS560 at the concentrations of 0.1 and 1 µM were able to significantly reduce the increased levels of these proinflammatory cytokines induced by LPS treatment (Figure 8). BS560 reduced all the genes more efficiently than the other compounds.

**FIGURE 9:**
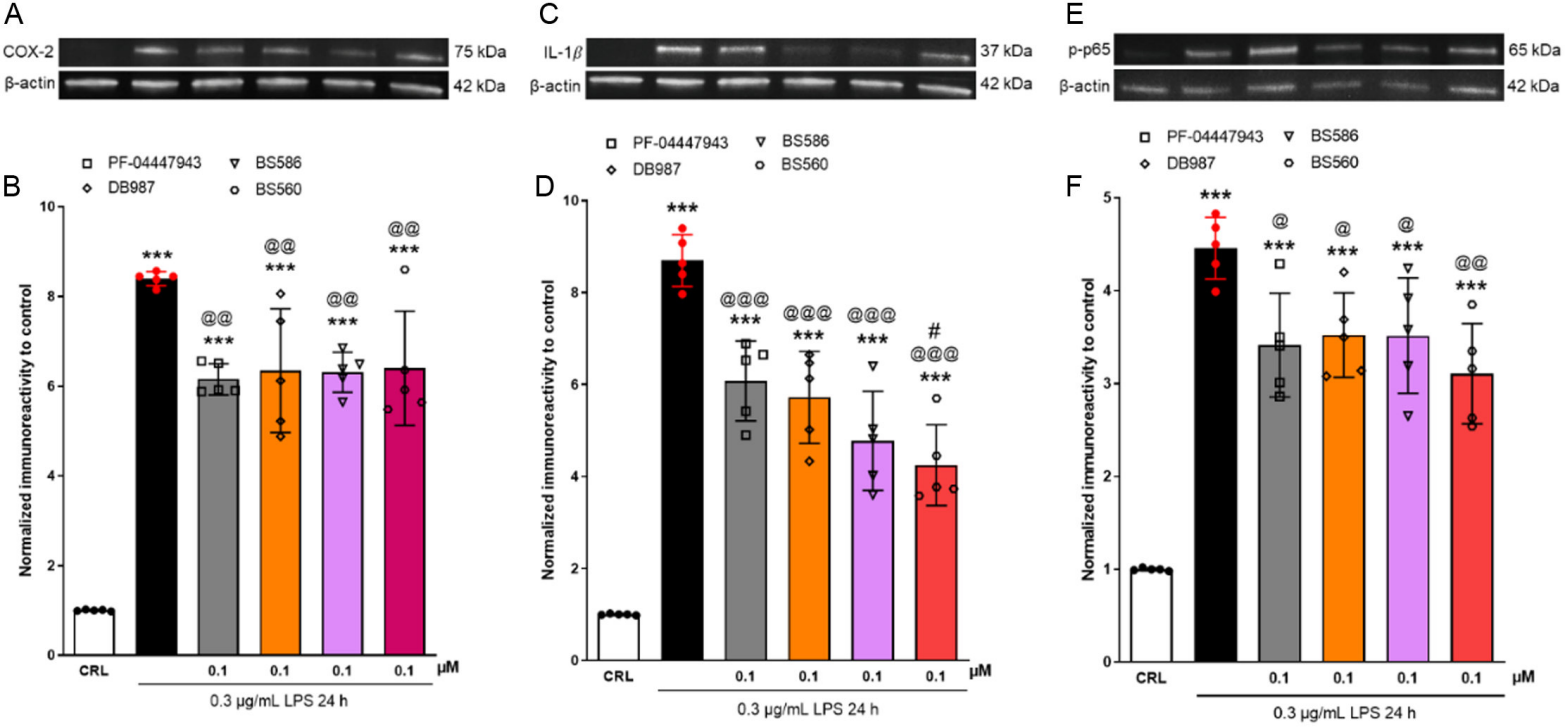
The effects of PDE9A inhibitors on proinflammatory proteins in rat mixed glial cells exposed to LPS. Cells were exposed to 0.3 µg/mL for 24 h and then processed for WB. The compounds were present in the incubation medium during LPS exposure. (A,C,E) Representative blots for COX‐2, IL‐1β, p‐p65, or β‐actin. (B,D,F) The quantitative analysis of immunoreactive bands shows that PDE9A inhibitors are able to reduce the increased levels of proinflammatory protein induced by LPS. Bars represent the mean ± SD from five independent experiments from different glial cell preparations, and each dot is the pool of two wells. ***p < 0.001 versus CRL; @ *p* < 0.05, @@ *p* < 0.01, and @@@ *p* < 0.001 versus LPS; # *p* < 0.05 versus LPS + PF‐04447943 (ANOVA + Tukey’s w test).

Given the modifications observed at the gene level, we also investigated whether these translated into protein modifications by analyzing some proinflammatory proteins, such as IL‐1β, COX‐2, and p‐p65. In this model of inflammation in vitro, the increased level of these proinflammatory proteins was significantly reversed by the treatment with PDE9A inhibitors, especially by BS560 (Figure 9).

AD is associated with mitochondrial dysfunction and the production of reactive oxygen species [44]. The exposure of astrocytes to Aβ results in a significant increase in mitochondrial reactive oxygen species (mtROS) production, which contributes to the pathogenesis of mitochondrial dysfunction and neuroinflammation in the brain [45]. Mitochondrial dysfunction in microglia and astrocytes also triggers and sustains neuroinflammation, and compromising their supportive role in these cells becomes an important key in the study of neurodegenerative diseases [46].

**FIGURE 10:**
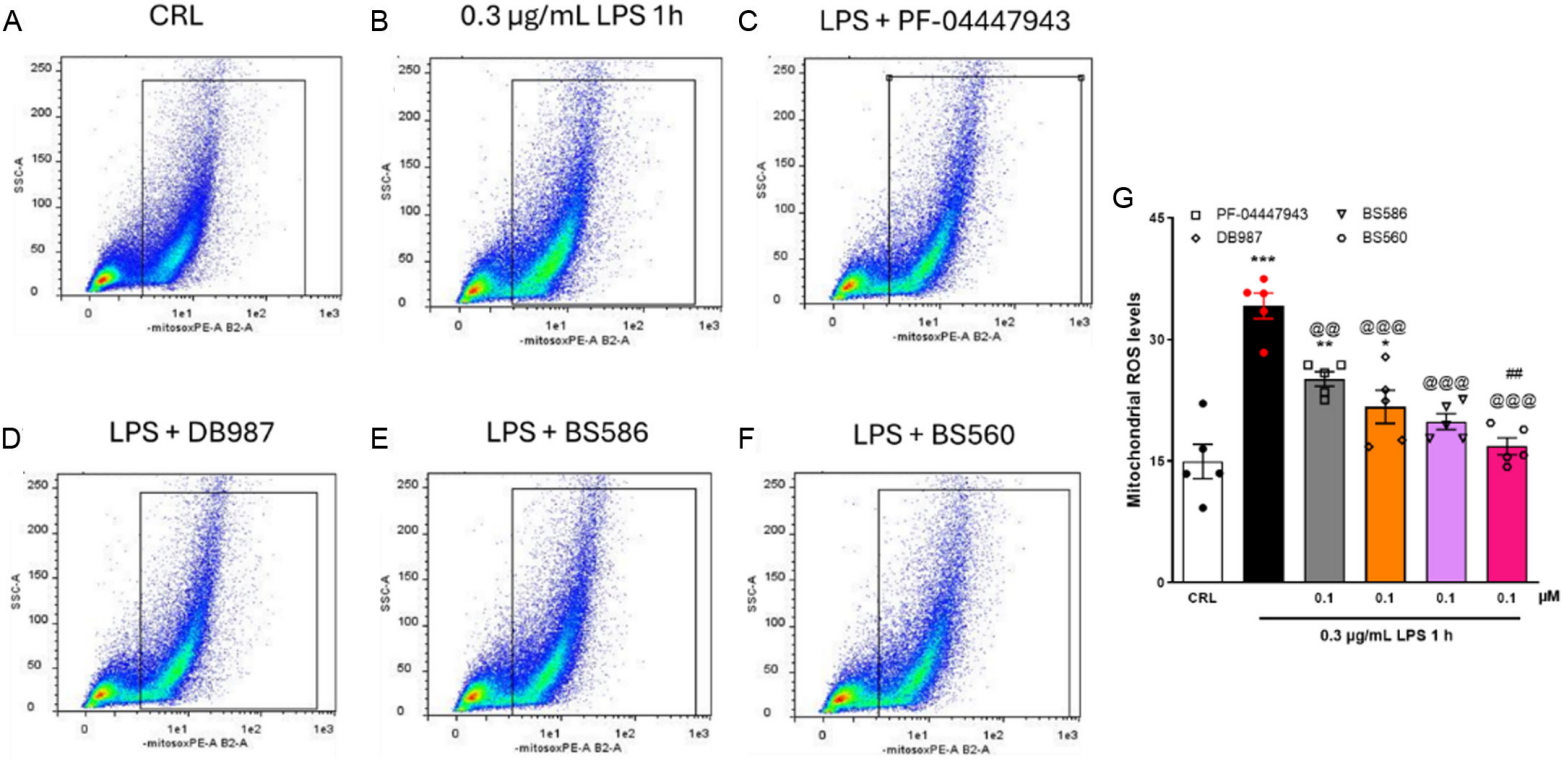
The effects of PDE9A inhibitors on mtROS production in glial cells exposed to LPS. Cells were exposed to 0.3 µg/mL LPS for 1 h and then processed for the quantification of mtROS by cytofluorimetric analysis. Compounds were present in the incubation medium during LPS exposure. (A–F) representative plots showing the amount of ROS produced during the treatments. The red color represents the highest density within a population. With decreasing density, the color transitions from yellow to green to blue. (G) Quantitative analysis of plots, showing that PDE9A inhibitors can significantly reduce the increased levels of mtROS induced by 0.3 µg/mL LPS for 1 h. Dots show the results of five experiments. *p < 0.05, **p < 0.01 and ***p < 0.001 versus CRL; @@ *p* < 0.01 and @@@ *p* < 0.001 versus LPS; ## *p* < 0.01 versus LPS + PF‐04447943. (ANOVA + Tukey’s w‐test).

In this study, oxidative stress was evaluated by the quantification of mtROS levels in glial cells using the MitoSOXTM Red mitochondrial assay, which detected the superoxide in the mitochondria of live cells. The LPS treatment induces an increase in ROS production, and this effect was reversed by the coincubation with PDE9A inhibitors, as shown in Figure 10. In this context, BS560 results was the most active compound.

Thus, these experimental findings are in line with computational and enzymatic data, but are currently limited to in vitro evidence, and further studies would be needed to evaluate the selectivity within the PDE family and to assess involved biochemical pathways.

## 3 Conclusion

This study combined computational and in vitro methods to discover new PDE9A inhibitors with antineuroinflammatory effects. Starting from the known and recently published PDE9A inhibitor DB987, a substructure‐based screen filtered a library of 672 compounds to 27 candidates. These were examined using molecular docking and MD simulations, which identified BS560 (6‐bromoquercetin) as the most promising due to its stable interactions with the key residues Gln453, Phe456, and Tyr424, reported to be pivotal for inhibition and selectivity. This computational prediction was validated in vitro using a newly developed HPLC‐based PDE9A assay, in which BS560 exhibited inhibitory activity comparable to that of PF‐04447943 and DB987. In contrast, BS586 exhibited weaker activity despite favorable docking, underscoring the importance of dynamic binding analysis for predicting functional inhibition. Additionally, in vitro tests in mixed glial cells, in which PDE9 expression was increased by LPS stimulation, showed that none of the tested compounds had significant effects on cell viability or cytotoxicity. Notably, BS560 exhibited the strongest anti‐inflammatory activity, significantly reducing COX‐2, IL‐1β, TNF‐α, and IL‐6 at both mRNA and protein levels, and reducing mitochondrial ROS production, indicating antioxidative and neuroprotective properties.

Overall, BS560 stood out as a promising candidate, combining potent PDE9A inhibition, low toxicity, and anti‐inflammatory and antioxidant effects. In this context, our results further underline the potential of the class of flavonoids, including isoflavones as the lead molecule DB987 and flavones as the newly identified BS560, as PDE inhibitors, prompting the community to further explore these molecules as PDE9A‐targeting agents.

## 4 Experimental Section

### 4.1 Substructure Search

An in‐house database comprising 672 synthesized compounds was used to identify novel PDE9A inhibitors. Subsequently, substructure‐based screening was conducted using six generated query structures (A‐F), yielding 27 compounds.

### 4.2 Molecular Docking

The complex of human PDE9A and its inhibitor PF‐04447943 was retrieved from the RCSB PDB (http://www.rcsb.org, access date 29 April 2025) with the PDB ID 4E90 (Resolution 2.50 Å). Before molecular docking analysis, the receptor was prepared through the Protein Preparation Workflow tool [47]. This step involved adding hydrogens, assigning disulfide bonds, adding missing side chains and loops using Prime, removing crystallographic waters, adjusting charges, and minimizing the convergence of heavy atoms to RMSD (value of 0.3 Å) under the OPLS4 force field. Then, ligands were prepared for docking using the LigPrep tool under the OPLS4 force field at target pH 7.4. Possible ionization and metal‐binding states were generated using an Epik ionizer. The binding poses of PF‐04447943 and DB987 in complex with PDE9A were obtained by molecular docking calculations using Glide as described in our previous study [20]. Then, the best pose of the PDE9A‐cognate ligand was evaluated by means of the RMSD value, for which a score under 2.50 Å is considered sufficient to consider the analysis accurate. For PDE9A/PF‐04447943, an RMSD of 1.18 Å was obtained, validating the following analyses. To rank docked ligands, the docking score parameter (expressed in kcal/mol) was used, and the best PDE9A‐binders were inspected from the point of view of interaction by considering PDE9A‐selective residues (Gln453, Phe456, and Tyr424) within 5.0 Å in the binding pocket.

### 4.3 MD Simulations

MD simulations were performed using the GPU‐accelerated Desmond tool of Schrödinger LLC (Schrödinger Release 2025‐1: Desmond MD System, D. E. Shaw Research, New York, NY, USA, 2021. Maestro‐Desmond Interoperability Tools, Schrödinger, New York, NY, USA 2021). Best identified ligand/PDE9A complexes from the SP molecular docking step that satisfied interaction and docking‐score requirements were used to prepare the solvated system for MD. Using System Builder, all three complexes were solvated with the explicit TIP3P water model. Periodic boundary conditions were set with an orthorhombic box shape of 10 × 10 × 10 Å. During ion placement, Na^+^ ions were used to neutralize the system; a concentration of 0.15 M of NaCl was set. A 500 ns run was performed for each complex under the OPLS4 force field, with a temperature of 300 K and a pressure of 1.0 bar. Further, the Martyna−Tuckerman−Klein chain coupling scheme with an isotropic coupling constant of 2.0 ps for the pressure control and the Nosé−Hoover chain coupling scheme for the temperature control (NPT ensemble) were used. The cutoff radius in the Coulomb interactions was 9.0 Å. A RESPA integrator was set with a time step of 2.0, 2.0, and 6.0 fs, respectively, for bonded interactions and near and far interactions. Trajectories were saved at 500 ps intervals for analysis. To analyze interactions between ligands and PDE9A, the Simulation Interaction Diagram tool and RMSD/RMSF evaluation were used. When ligand−protein contacts were evaluated, a contact strength threshold value of 30% was considered for all of the analyses. Artworks were made with Schrödinger Release 2025‐2: Maestro, Schrödinger, LLC, New York, NY, 2025.

### 4.4 ADME Prediction

For the studied compounds, physicochemical descriptors relevant for drug‐likeness, ADME parameters and pharmacokinetic properties were predicted using the SwissADME tool (www.swissadme.ch) [48]. In particular, parameters related to Lipinski’s rule were considered [49].

### 4.5 HPLC‐Based Enzymatic Assay

The enzymatic activity assay was performed by HPLC using a Pro‐Star system (Palo Alto, CA) equipped with a 1706 UV–vis detector (Bio‐Rad, Hercules, CA) and a C‐18 column (5 μm, 4.6 × 150 mm) (Agilent, Santa Clara, CA). An appropriate gradient of 0.1% formic acid (A) and acetonitrile (B) was used as the mobile phase with an overall flow rate of 1 mL min^−1^, and the detection wavelength was set to 254 nm. The general method for the analyses is reported in the following: 0 min (95% A–5% B), 0.5 min (95% A–5% B), 4 min (75% A–25% B), 4.5 min (5% A–95% B), 9 min (5% A–95% B), and 10 min (95% A–5% B). This method successfully differentiated cGMP and GMP peaks. The samples were prepared as follows: 120 μL total volume with Tris HCl and MgCl_2_ buffer (pH 7.4; 20 mM), ligand (27.5 μM, from a 10 mM DMSO stock), cGMP (27.5 μM, from a 10 mM LC‐MS grade water stock), and PDE9A (12.5 nM). PDE9A (SRP0274), buffers, and reagents for HPLC tests are >95% pure by HPLC and were purchased from Merck (Darmstadt, Germany).

The analyses were performed in triplicate with 4 different time points: *t*_0_ (0 min), *t*_1_ (60 min), *t*_2_ (120 min), and *t*_3_ (240 min) and the temperature of the vials was kept constant at 37 °C with a stirring hot plate.The change of the area of the cGMP peak was used as reference to follow the enzymatic reaction over time. To further confirm the occurrence of the reaction, it was also noted that the appearance of the GMP peak upon the decline of the cGMP one. To evaluate how the different ligands could alter the half‐life of the reaction (*t*_1/2_) of cGMP conversion by PDE9A, an exponential one‐phase decay model (*Y *= (*Y*_0_ − plateau)*exp(−*K* ^*^ *X*) + plateau) was applied using GraphPad Prism v. 8 for Windows (GraphPad Software, San Diego, CA). *Y*_0_ is the *Y* value when *X* is 0. Plateau represents the *Y* value at infinite times, and was constrained to a constant value of 0. *K* is the rate constant, expressed in reciprocal minutes. The *t*_1/2_ was calculated as equal to $\frac{\ln\;(2)}{K}$ [50]. Chromatograms raw files in *.cdf* format were obtained using Varian Star Chromatography Workstation 6.30 for Windows, Varian, Inc. (acquired by Agilent Technologies in May 2010; Walnut Creek, CA). Chromatograms were processed using RStudio 2025.5.1.513, Posit team (2025). RStudio: Integrated Development Environment for R (Posit Software, PBC, Boston, MA; http://www.posit.co/). In particular, *ncdf4* package was used to extract raw absorbance signals from the files, which were aligned to time vectors using the HPLC run length and sampling intervals [51]. Baseline correction was performed using the asymmetric least squares (ALS) algorithm implemented in the baseline R package [52]. The obtained signals were smoothed with a symmetric moving average filter with a window size (*k*) of 11 data points, using the rollmean function from the zoo package [53]. Peaks were identified using the findpeaks function from the pracma package. Peak boundaries were established with a threshold‐based extension algorithm. More in detail, the boundaries were extended from the apex to the left and right until the signals fell below the corrected baseline, defined as the global median of the chromatographic signal. Peak area were calculated using the trapezoidal integration method (trapz function in pracma package) [54]. Chromatogram plots were generated using *ggplot2* with peak areas highlighted in different colors.

### 4.6 Animals

Wistar rats (Charles River, Italy) were used for the preparation of primary cultures of mixed glial cells. All animal experiments satisfied the regulatory requirements of the European Parliament (Directive 2010/63/EU), European Union Council (September 22, 2010), and Italian Animal Welfare Law (DL 26/2014). The protocol received approval from the Institutional Animal Care and Use Committee (Univ. Florence: project n. 17E9C.N.0Y0 and 17E9C.N.7J7) and the Italian Ministry of Health. Animal suffering and the animal number required for data reproducibility were minimized.

### 4.7 Mixed Rat Glial Cells

Primary mixed cortical cultures of astrocytes and microglia (referred as “mixed glial cells”) were prepared from postnatal day 1–2 Wistar rats pups of both sexes as previously described [55, 56] and grown in Dulbecco’s Modified Eagle’s Medium (DMEM)—high glucose DMEM D6546‐500 ML, Merck Life Science, Milan, Italy) + 10% fetal bovine serum (FBS) (Honduras Origin F7524, Merck Life Science, Milan, Italy). Briefly, cortices were isolated in cold phosphate‐buffered saline (PBS) and then incubated for 10 min at 37 °C in PBS containing 0.25% trypsin (Trypsin‐EDTA Solution T3924‐100 ML, Merck Life Science, Milan, Italy) and 0.05% DNase (Deoxyribonuclease I from bovine pancreas DN25‐100 MG, Merck Life Science, Milan, Italy). After blocking enzymatic digestion with the addition of 10% heat‐inactivated FBS, cortices were mechanically disrupted, washed, and the cells resuspended in DMEM + 10% FBS and plated in 24‐well plates. The cells after they had reached the confluence were stimulated with 0.3 μg/mL bacterial LPS (Lipopolysaccharides from Escherichia coli O111:B4 L3012‐5 MG Merck Life Science, Milan, Italy). PDE9A inhibitors were added to the culture medium together with LPS. Cells were lysed after 6 h of exposure for real‐time PCR and after 24 h for Western Blot assays. Cultures were incubated at 37 °C, 100% humidity, and 95% air/5% CO_2_ atmosphere and used for experiments at days 7–8 in vitro.

### 4.8 Quantitative Real‐Time Polymerase Chain Reaction (RT‐qPCR)

Evaluation of Cyclooxygenase‐2 (COX‐2), IL‐1β, IL‐6, and TTNFα Gene Expression by Quantitative Real‐Time Polymerase Chain Reaction (RT‐qPCR). Total RNA was isolated from mixed glial cells using Trizol Reagent REF #15596026 (Thermo Fisher Scientific, Milan, Italy). One μg of RNA was retrotranscribed using PrimeScript RT Reagent Kit with Gdna Eraser (Perfect Real Time 100 Rxns #RR047A, Diatech Lab Line Srl, Jesi,AN, Italy). RT‐qPCR was performed as reported [57]. Primers were purchased from Integrated DNA Technologies (Iowa, USA). Ribosomal 18S RNA was used as the normalizer. Quantitative PCR was performed using the following procedure: 95 °C for 30 s, 95 °C for 5 s and 60 °C for 15 s for 45 cycles. The sequences of the primers used read as follows: PDE 9A (PDE9A) rat forward 5′‐TCAGAGCGAACTCCCTACAAGGTGAG‐3′ and reverse 5′‐CTCCACCACTTTGAGTCCTTCCAATTCC‐3′; COX‐2 rat forward 5′‐GATGACGAGCGACTGTTCCA‐3′ and reverse 5′–TGGTAACCGCTCAGGTGTTG–3′; IL‐1β rat forward 5′‐AATGCCTCGTGCTGTCTGACCC‐3′ and reverse 5′‐TGGGTGTGCCGTCTTTCATCAC‐3′; TNF‐α rat forward 5′‐GCCAATGGCATGGATCTCAAAG‐3′ and reverse 5′‐AGGGCTCTTGATGGCAGAGAGG‐3′; IL‐6 rat forward 5′‐GTCAACTCCATCTGCCCTTCA‐5′ and reverse 5′–GAAGGCAACTGGCTGGAAGT‐3′;18S rat forward 5′‐GGCGGCTTTGGTGACTCTAGATAACC‐3′ and reverse 5′‐CCTGCTGCCTTCCTTGGATGTGG‐3′.

### 4.9 Western Blotting

Evaluation of COX‐2, IL–1β, and Phospho Nuclear Factor NF‍‐‍Kappa‐B p65 Subunit (p‐p65) protein expression by Western Blotting. Mixed glial cells were washed with cold 0.01 M PBS, pH 7.4, at the end of the treatment and dissolved in 1% SDS. The BCA (bicinchoninic acid) protein assay was used to quantify the total protein levels. Lysates (20 μg/lane of protein) were resolved by electrophoresis on a 4%–20% SDS‐polyacrylamide gel (Bio‐Rad Laboratories, Hercules, CA) and transferred onto nitrocellulose membranes. Blots were blocked for 1 h at room temperature in 20 mM Tris‐buffered saline, pH 7.6, 0.1% Tween 20 (TBS‐T) containing 5% nonfat dry milk, and then incubated overnight at 4 °C with rabbit polyclonal antibodies against COX‐2 (# ab15191) and IL‐1β (# ab9722) (all from Abcam, Cambridge CB2 0AX, UK), PDE9A (#ABN32) from Sigma (St. Louis, MO), monoclonal rabbit antibodies against Phospho‐NF‐κB p65 (Ser536) (93H1) (#3033) (Cell Signaling Technology, Beverly, MA), diluted 1:1000 in TBS‐T containing 5% bovine serum albumin. As a loading control, the monoclonal anti‐β‐actin antibody (#A3854) was from Sigma (St. Louis, MO). Immunodetection was performed with secondary antibodies (1:3000 antimouse or antirabbit IgG from donkey, Amersham Biosciences, Amersham, UK) conjugated to horseradish peroxidase in TBS‐T containing 5% nonfat dry milk. Membranes were washed with TBS‐T, and then reactive bands were detected using chemiluminescence (ECLplus; Euroclone, Padova, Italy). Quantitative analysis was performed using the QuantityOne analysis software (Bio‐Rad, Hercules, CA).

### 4.10 Cytotoxicity by 3‐(4,5‐dimethylthiazol‐2‐yl)‐2,5‐diphenyltetrazolium Bromide (MTT) Assay

To assess cell viability after PDE9A inhibitor treatment, the MTT assay was performed in rat mixed glial cells [58]. Cells were seeded in a 24‐well plate and grown at 37 °C in an atmosphere of 5% CO_2_ in DMEM medium. When the cells were approximately 70%–80% confluent, they were exposed for 24 h to PDE9A inhibitors alone at the concentrations of 0.1 and 1 µM. In other experimental set, cells were exposed to 0.3 µg/mL of LPS and PDE9A inhibitors. The medium of each well was separated from the cells and stored for LDH assay, and cells were treated with 1 mg/mL of MTT for 15 min at 37 °C and 5% CO_2_. Finally, DMSO was added to dissolve MTT formation and absorbance was measured at 550 and 690 nm. Cell viability was expressed as a percentage compared to the cells incubated only with DMEM medium (CRL). Triton X‐100 was employed in the MTT assay as the negative control since its detergent action disrupts the cells.

### 4.11 LDH Assay

Cytotoxicity after ligand exposure was verified with the LDH assay. Damage in mixed glial cells was quantitatively assessed by measuring the amount of LDH released by the damaged cells into the culture medium, 24 h after PDE9A inhibitors alone or in combination with LPS exposure, by adding catalyst and dye solutions of a Cytotoxicity Detection Kit (LDH) (Roche Diagnostics, Indianapolis, IN). The absorbance values were recorded at 490 and 690 nm. Cytotoxicity was expressed as a percentage compared to the maximum LDH release in the presence of Triton X‐100 (TX).

### 4.12 Evaluation of mtROS

As previously reported [59], the mixed glial cells were stained with MitoSOX at the final concentration of 2.5 μM for 15 min, then washed with PBS, detached with accutase, and resuspended with PBS. The stained cells were acquired using a MACSQuant Analyzer 10 Flow Cytometer (Miltenyi Biotec, Bergisch Gladbach, Germany), and the data were analyzed by Flowlogic (Miltenyi Biotec).

### 4.13 Statistical Analysis

For HPLC analysis, data are expressed as mean ± SD and were evaluated with unpaired Student’s t‐test. The statistical significance of differences between control and studied compounds was analyzed using one‐way ANOVA followed by a post hoc Dunnett’s test for multiple comparisons. GraphPad Prism v. 8 for Windows (GraphPad Software, San Diego, CA) was used to perform statistical analyses.

For in vitro studies, data are expressed as mean ± SD. Data on gene and protein expression levels of PDE9A were evaluated with an unpaired Student’s t‐test. The statistical significance of differences between control and treatment groups in MTT and LDH assays was analyzed using one‐way ANOVA followed by a post hoc Dunnett’s test for multiple comparisons. One‐way ANOVA followed by post hoc Tukey test for multiple comparisons was used to analyze mtROS and proinflammatory gene and protein expression levels. GraphPad Prism v. 8 for Windows (GraphPad Software, San Diego, CA) was used to perform all statistical analyses, considering a probability value (*p*) < 0.05 as significant.

## Author Contributions

All authors contributed to experiments, writing, and revising the paper.

## Conflicts of Interest

The authors declare no conflicts of interest.

## Supporting information

Supplementary Material

## Data Availability

The data that supports the findings of this study are available in the Supporting Information of this article.
