# Supplementary material for: Structure‐Guided Optimization of PDE9A Inhibitors Tackling Neuroinflammation: A Combined Computational and In Vitro Study
Source: ChemMedChem. 2026 Aug 31;21(17):e70461. doi: 10.1002/cmdc.70461 (PMC13529921; doi:10.1002/cmdc.70461)
Supplement: Supplementary file 1 [file CMDC-21-e70461-s001.pdf]

# - Supporting Information -

Structure-Guided Optimization of PDE9A Inhibitors Tackling Neuroinflammation: a Combined Computational and *In Vitro* Study

Giovanni Ribaudo[1,*,†], Elisa Landucci[2,†], Margrate Anyanwu[1], Costanza Mazzantini[2], Matteo Giannangeli[1], Stefano Rosa[1], Francesca Mainolfi[2], Maura Calvani[3], Maurizio Memo[1], Domenico E. Pellegrini-Giampietro[2] and Alessandra Gianoncelli[1,*]

[1] *Department of Molecular and Translational Medicine, University of Brescia, Viale Europa 11, 25123 Brescia, Italy*

[2] *Department of Health Sciences, Section of Clinical Pharmacology and Oncology, University of Florence, Viale Pieraccini 6, 50139 Florence, Italy*

[3] *Department of Pediatric Hematology-Oncology, Meyer Children's Hospital IRCCS, 50139 Florence, Italy*

Index of figures

Figure S1. 2D structures of 27 compounds identified from the in-house database screening using the six generated queries. 3

Figure S2. Docking interaction patterns of PDE9A in complex with DB987 (A), BS586 (B), and BS560 (C). 4

Figure S3. Interaction profiles deriving from MD simulation from Simulation Interaction Analysis of PDE9A complexes with BS586 (A) and BS560 (B), using a 30% threshold. 5

Figure S4. RMSF plots for BS586 (A) and BS560 (B). 6

Figure S5. Frames 1, 351, 701, and 950 extracted from MD simulation illustrating interactions between BS586 and PDE9A, with hydrogen bonds (yellow) and π–π stacking (light blue) involving key residues Tyr424, Gln453, and Phe456. 7

Figure S6. Frames 1, 351, 701, and 950 extracted from MD simulation illustrating interactions between BS560 and PDE9A, with hydrogen bonds (yellow) and π–π stacking (light blue) involving key residues Tyr424, Gln453, and Phe456. 8

Figure S7. Comparison between 4 chromatograms showing the peak of cGMP (red) at different time points. In this experiment, PDE9A was absent, and the integral remained stable throughout the experimental time. . 9

Figure S8. HPLC-based enzymatic assay conducted in the presence of PDE9A, which showed prompt conversion of cGMP (red) into GMP (yellow). 9

Figure S9. HPLC-based enzymatic assay conducted in the presence of PDE9A and of the inhibitor BS560, showing a reduced conversion of cGMP (red) into GMP (yellow). 10

Figure S10. Exponential one-phase decay of cGMP relative concentration over time in the presence of different inhibitors. 10

Figure S11. ADME prediction of compound BS560. 11

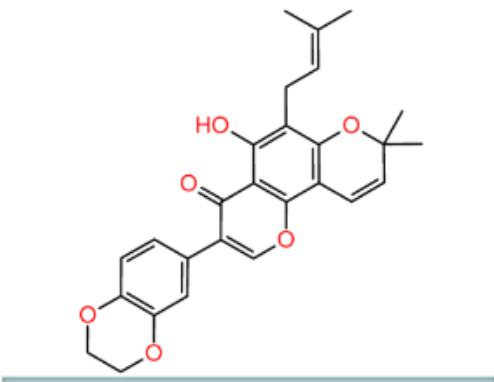

BS542

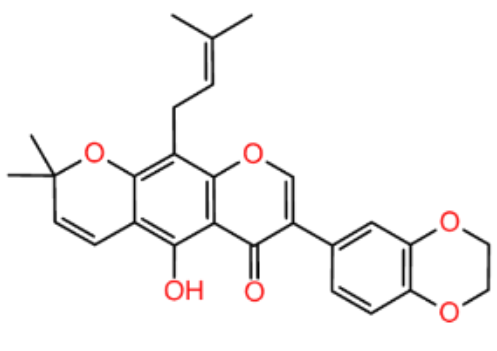

BS547

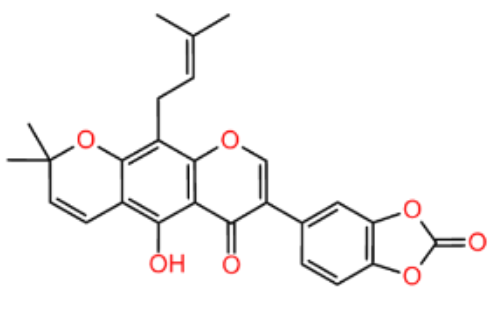

BS551

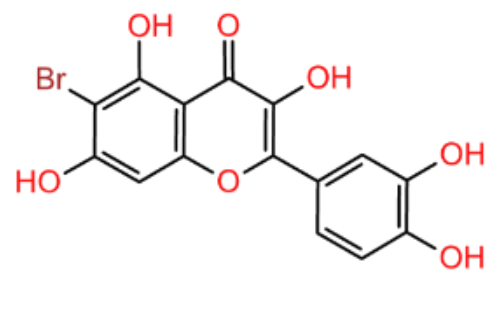

BS560

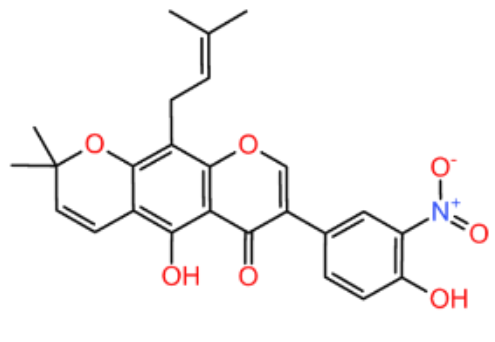

BS563

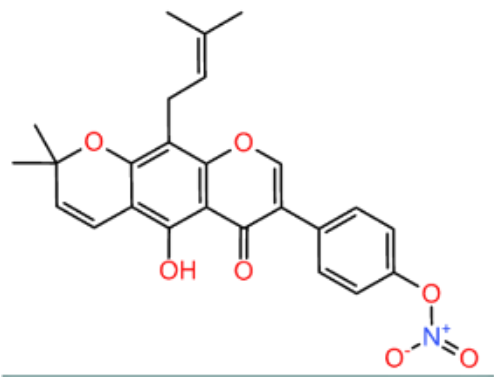

BS566

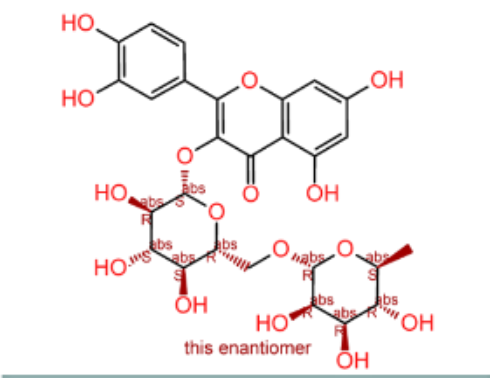

BS570

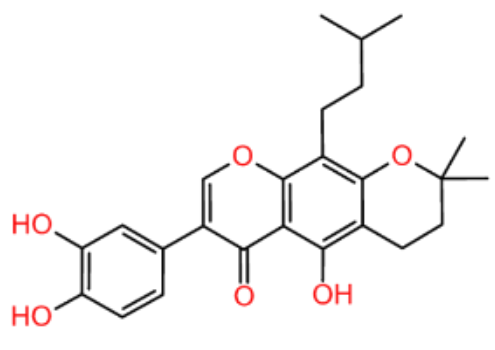

BS571

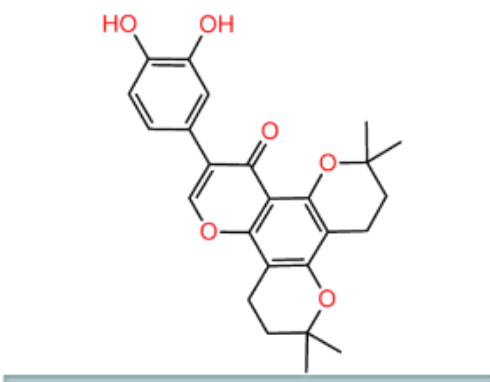

BS573

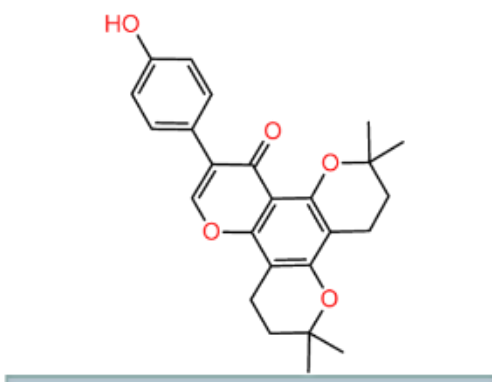

BS574

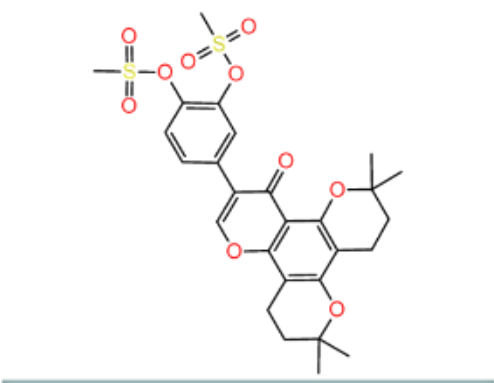

BS575

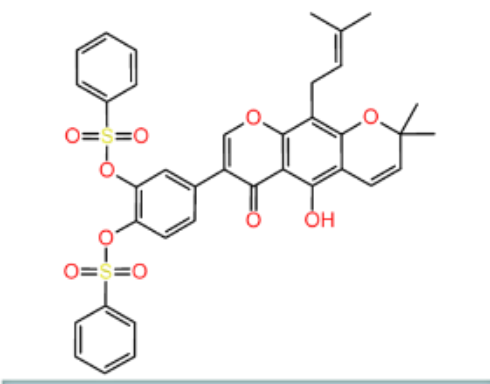

BS576

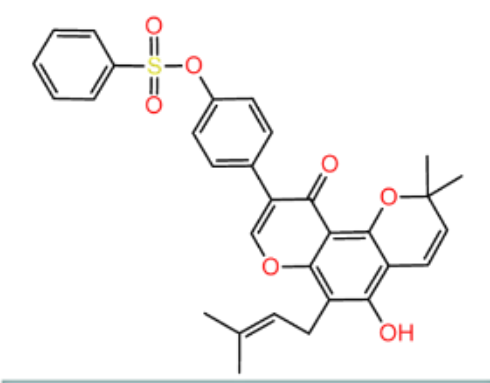

BS577

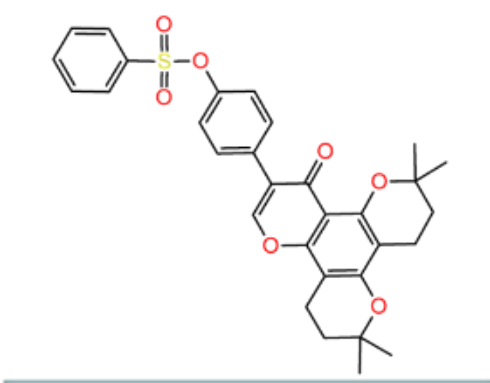

BS578

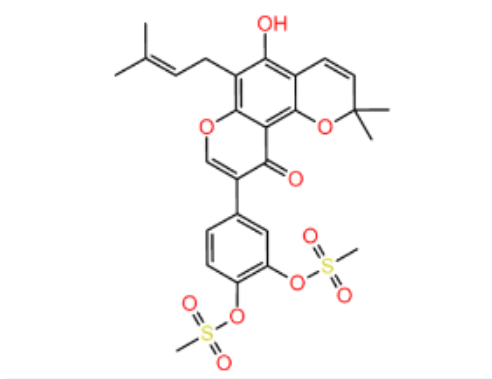

DB987

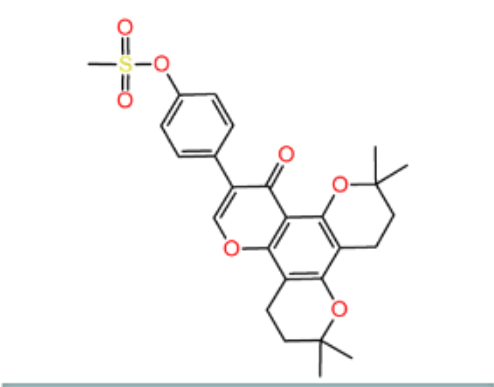

BS580

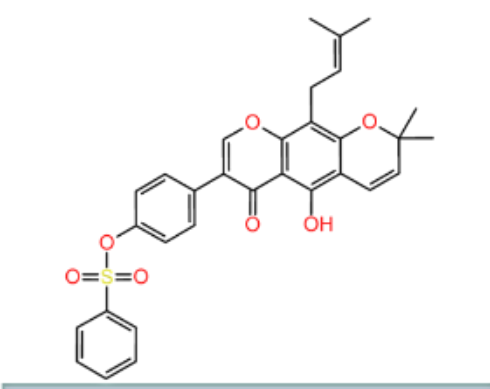

BS581

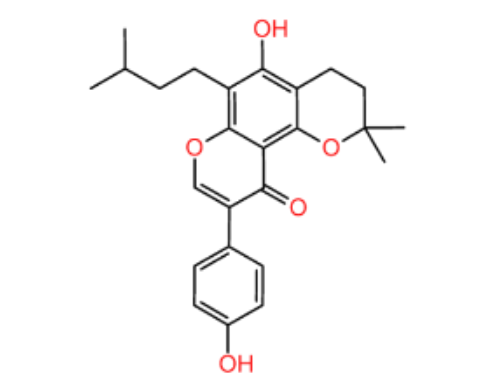

BS582

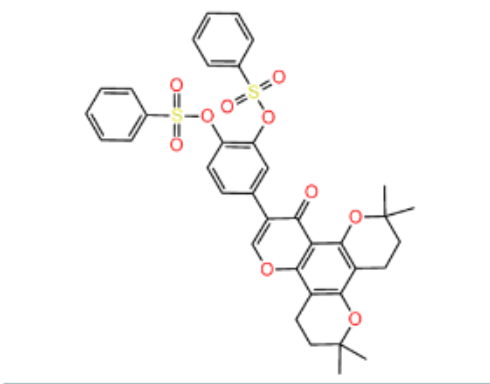

BS583

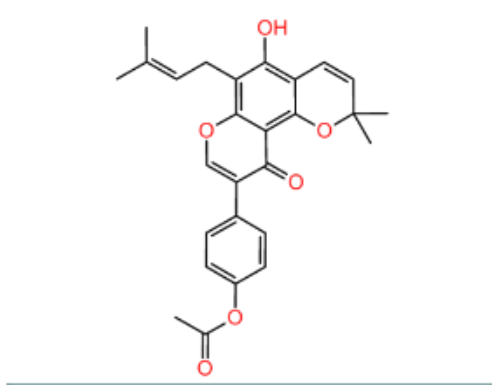

BS584

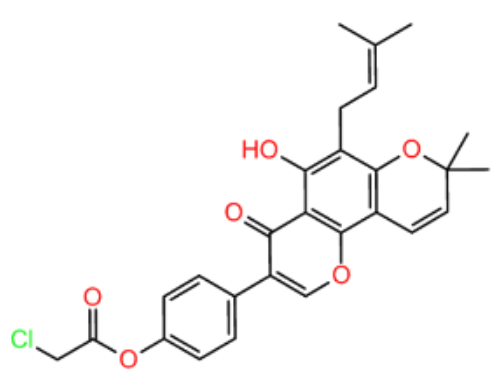

BS585

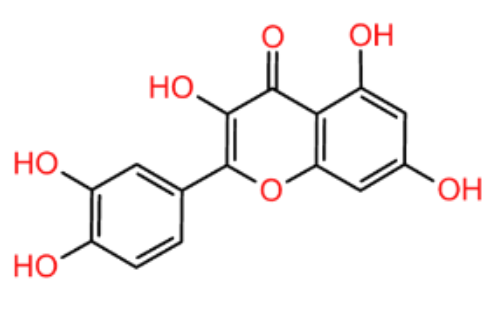

BS586

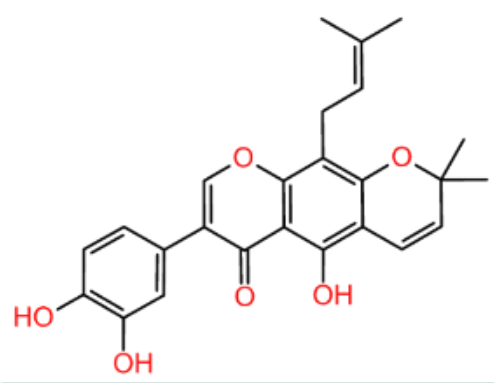

BS587

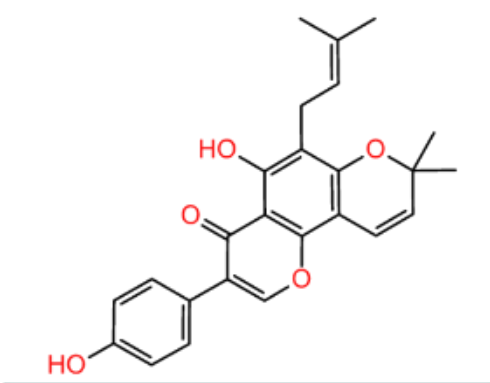

BS588

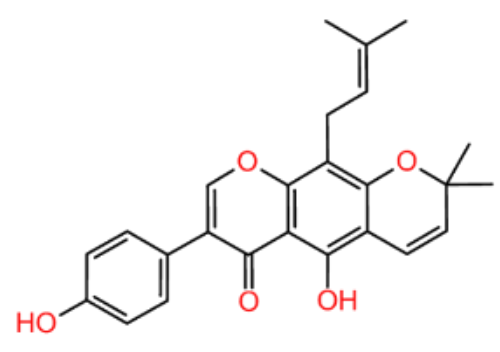

BS589

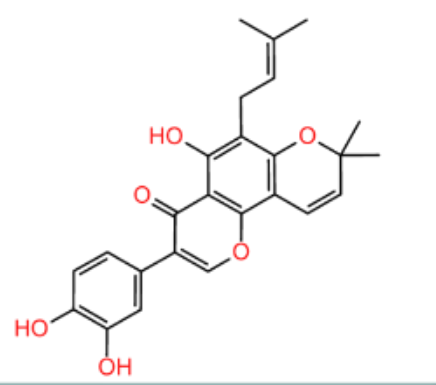

BS590

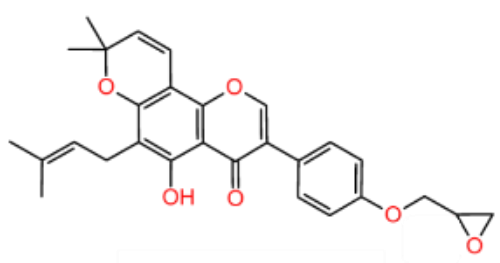

BS616

Figure S1. 2D structures of 27 compounds identified from the in-house database screening using the six generated queries.

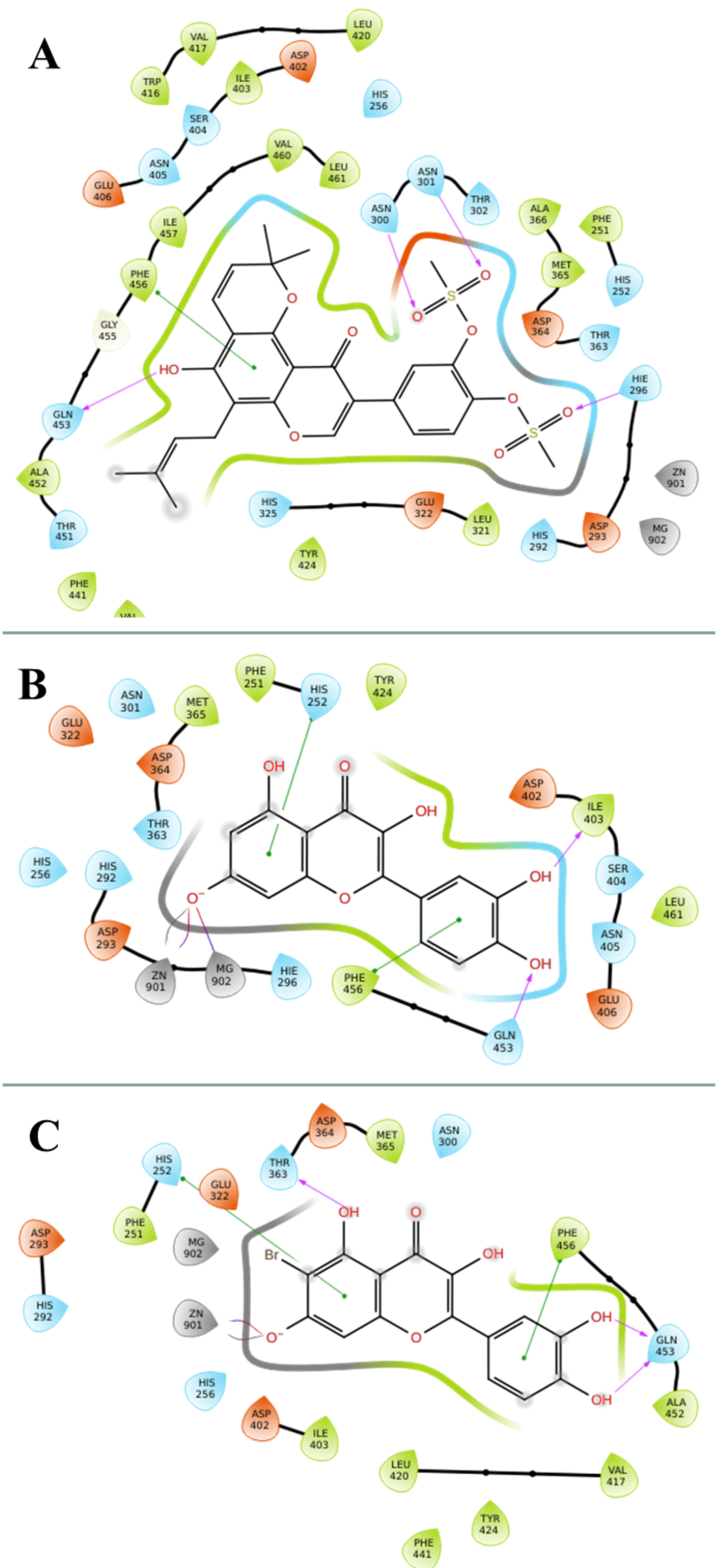

Figure S2. Docking interaction patterns of PDE9A in complex with DB987 (A), BS586 (B), and BS560 (C).

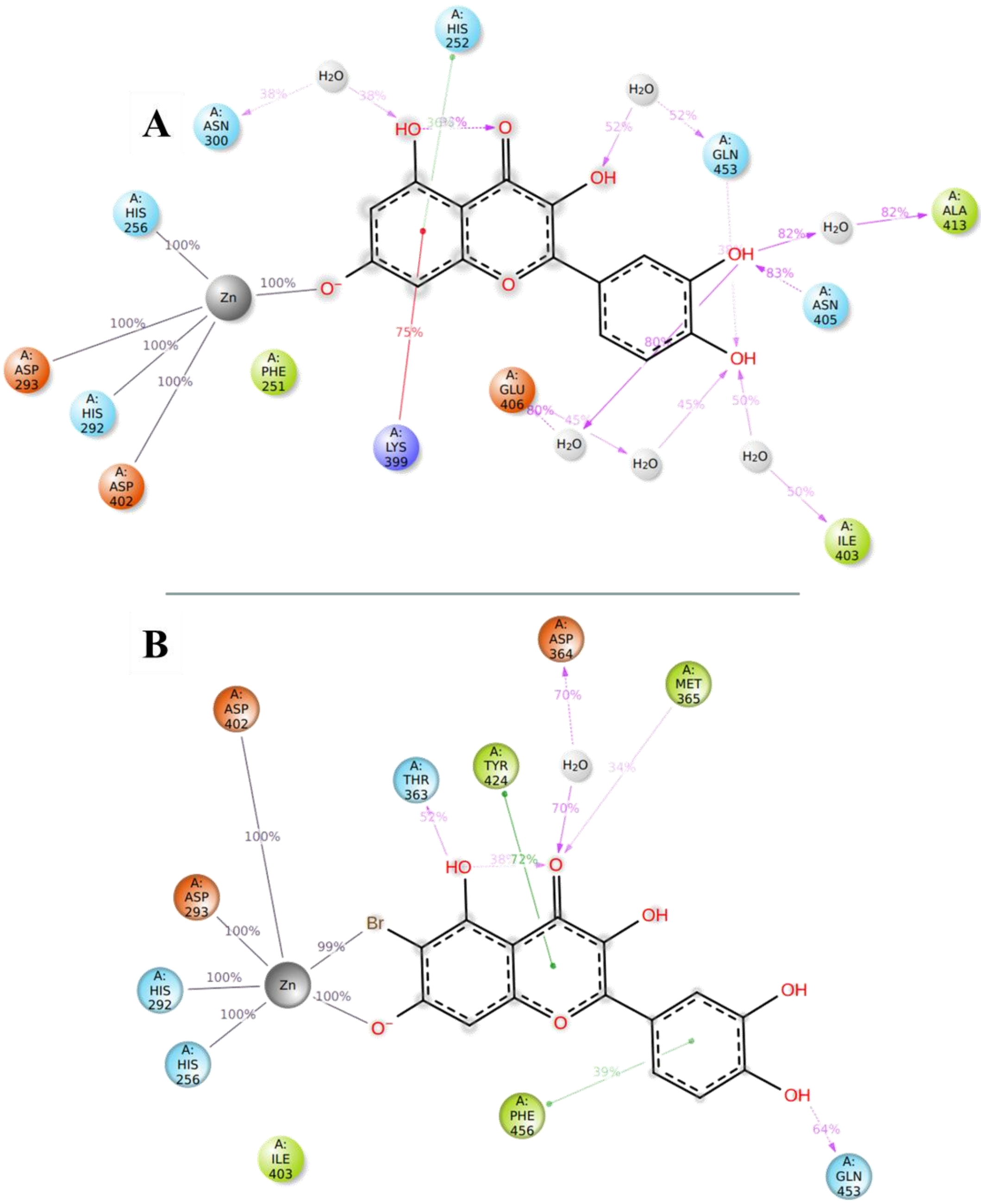

Figure S3. Interaction profiles deriving from MD simulation from Simulation Interaction Analysis of PDE9A complexes with BS586 (A) and BS560 (B), using a 30% threshold.

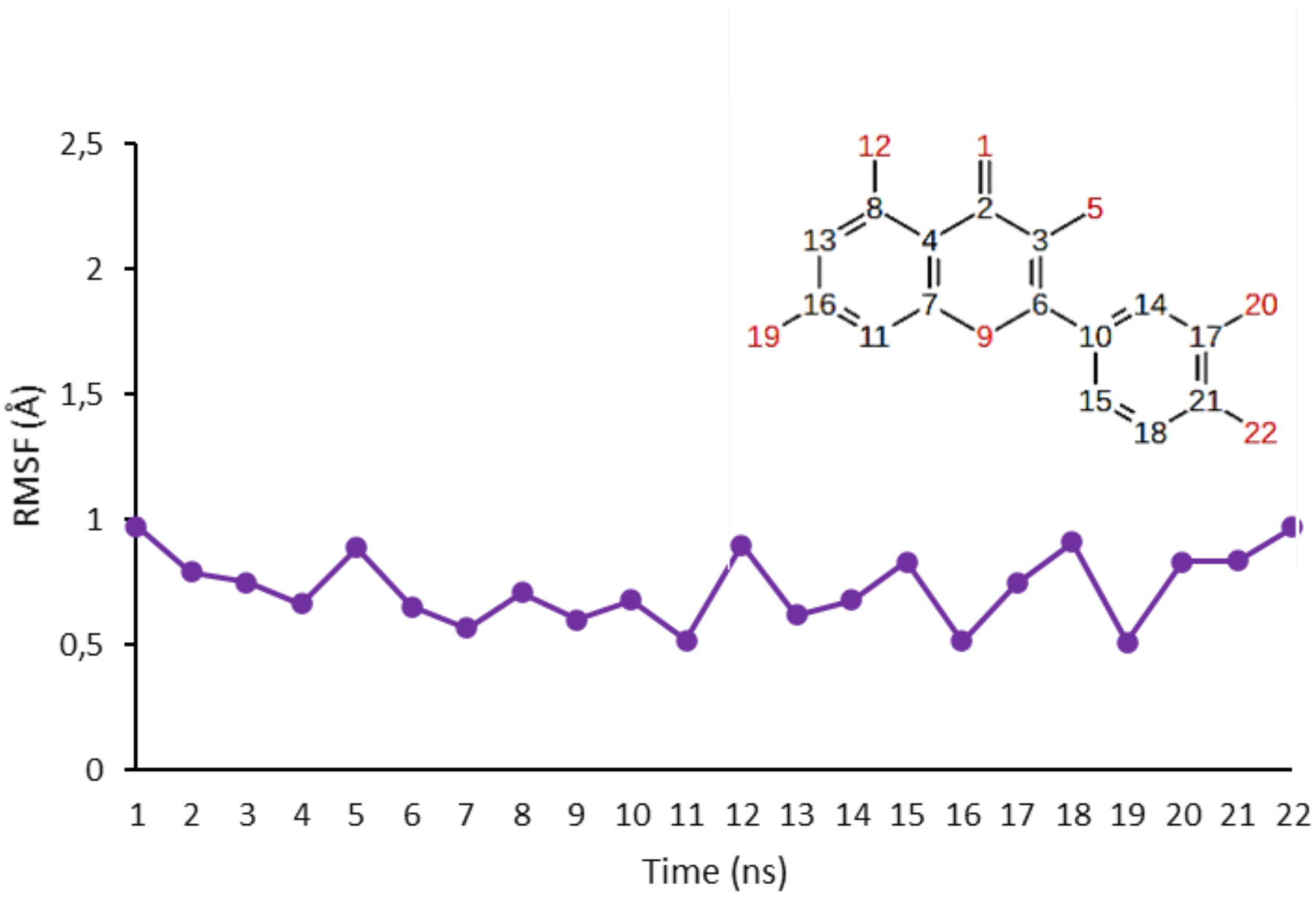

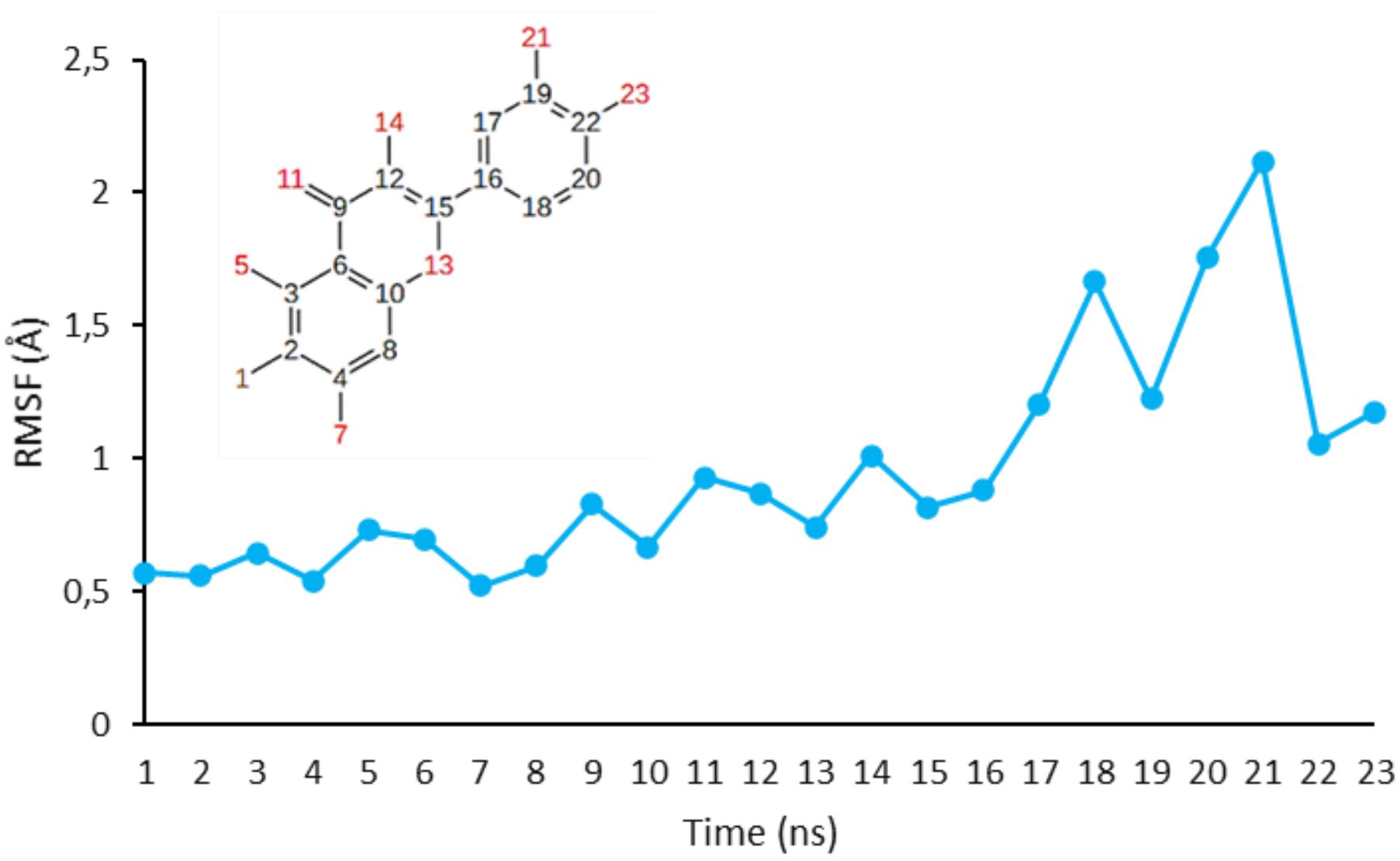

Figure S4. RMSF plots for BS586 (A) and BS560 (B).

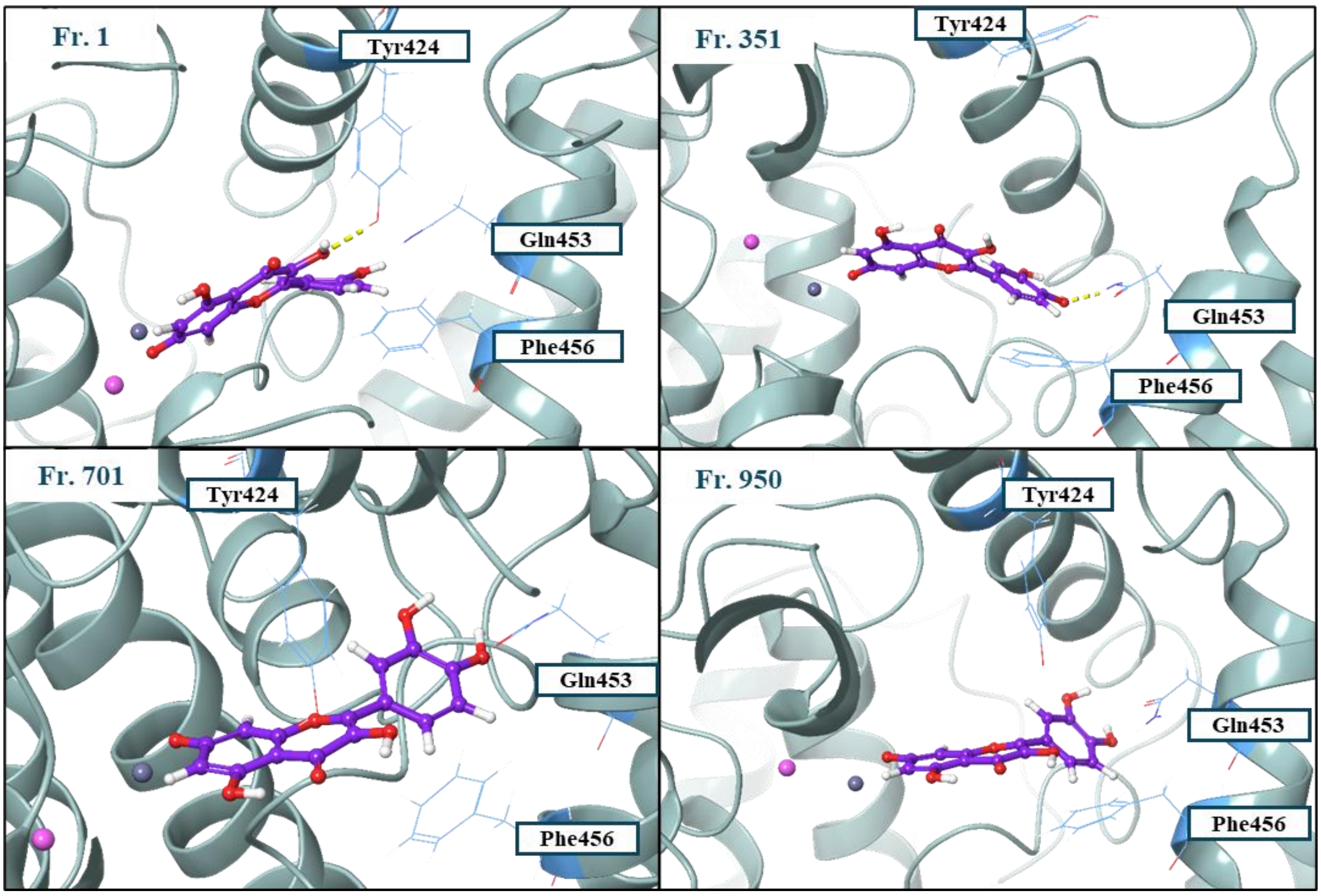

Figure S5. Frames 1, 351, 701, and 950 extracted from MD simulation illustrating interactions between BS586 and PDE9A, with hydrogen bonds (yellow) and π–π stacking (light blue) involving key residues Tyr424, Gln453, and Phe456.

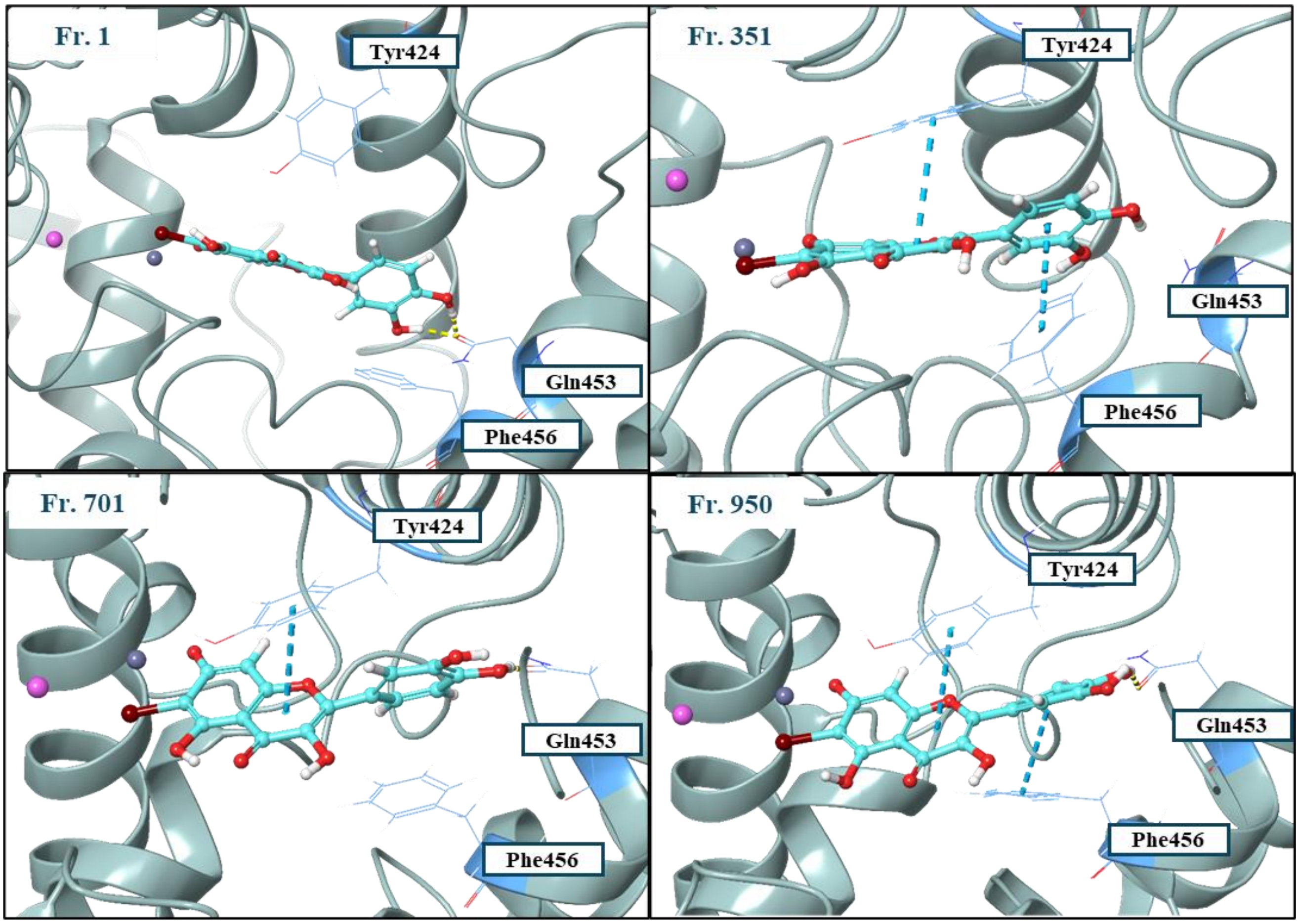

Figure S6. Frames 1, 351, 701, and 950 extracted from MD simulation illustrating interactions between BS560 and PDE9A, with hydrogen bonds (yellow) and π–π stacking (light blue) involving key residues Tyr424, Gln453, and Phe456.

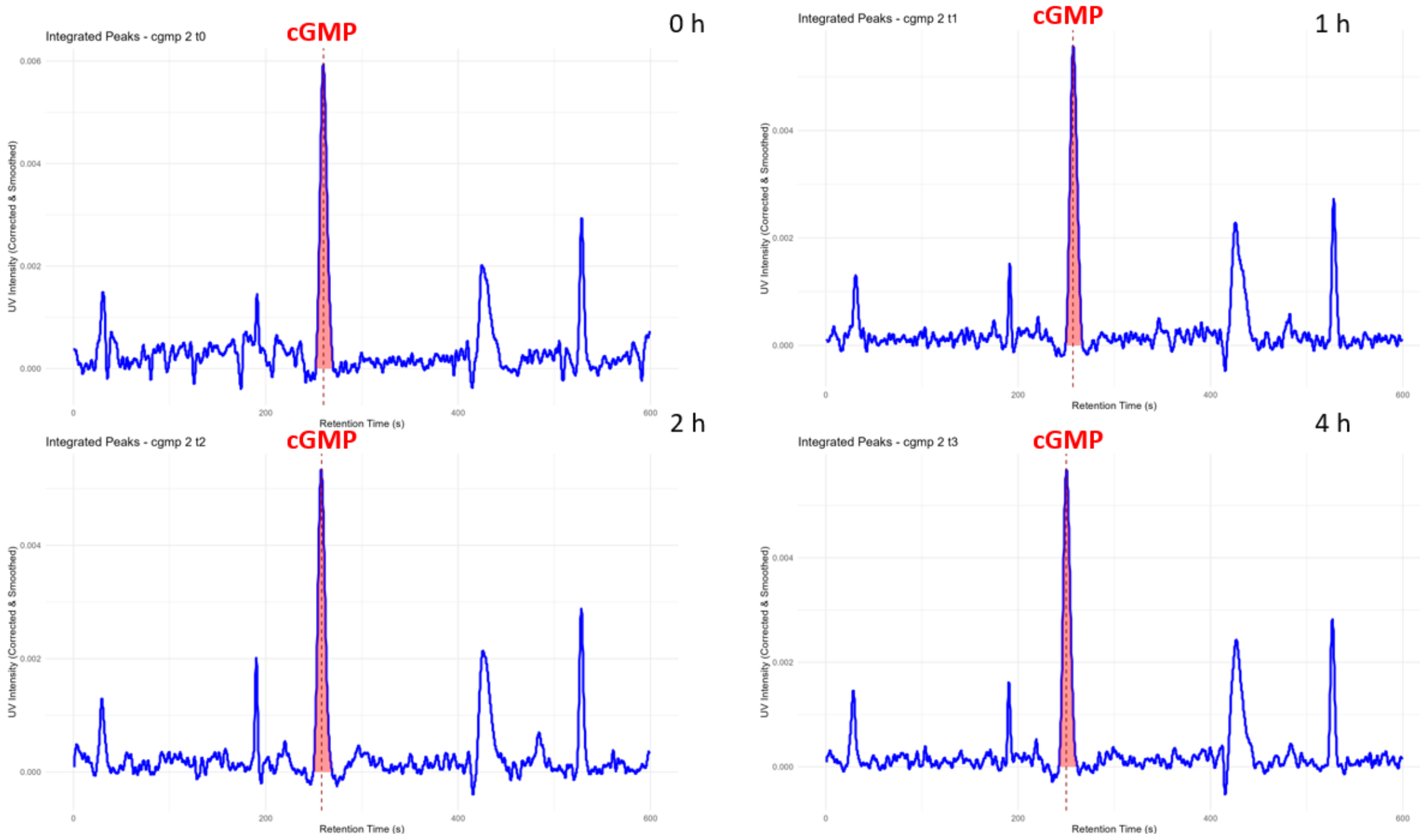

Figure S7. Comparison between 4 chromatograms showing the peak of cGMP (red) at different time points. In this experiment, PDE9A was absent, and the integral remained stable throughout the experimental time.

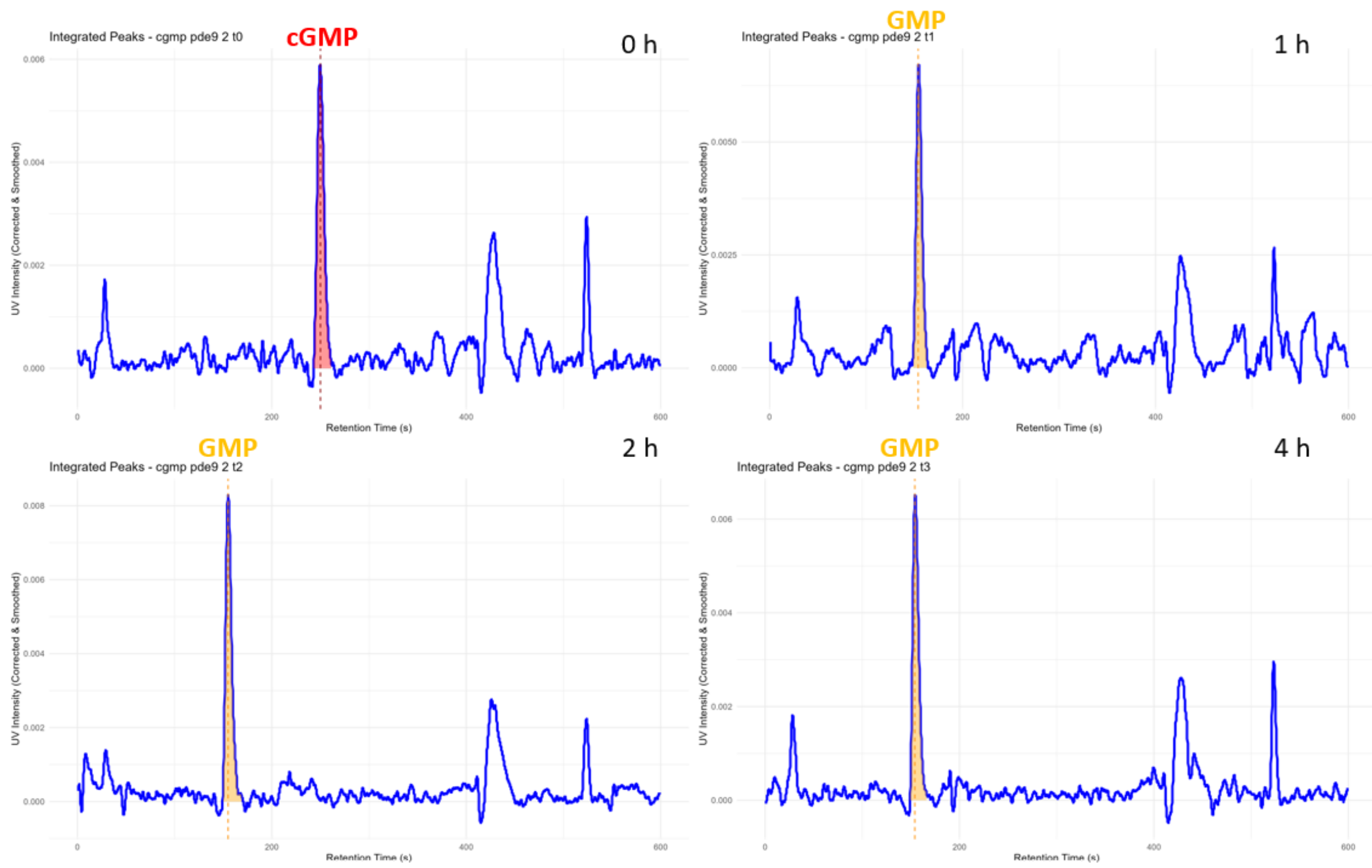

Figure S8. HPLC-based enzymatic assay conducted in the presence of PDE9A, which showed prompt conversion of cGMP (red) into GMP (yellow).

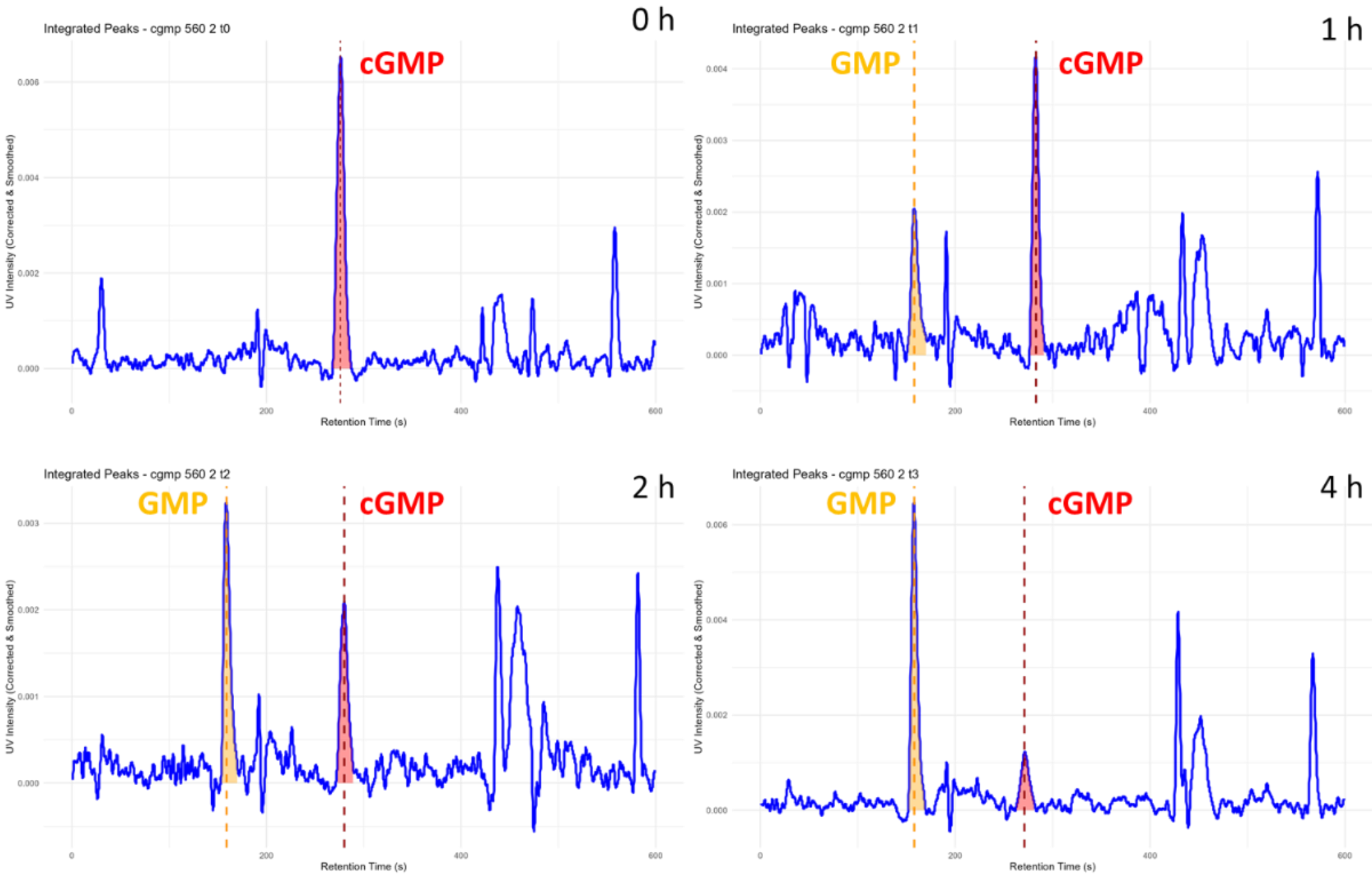

Figure S9. HPLC-based enzymatic assay conducted in the presence of PDE9A and of the inhibitor BS560, showing a reduced conversion of cGMP (red) into GMP (yellow).

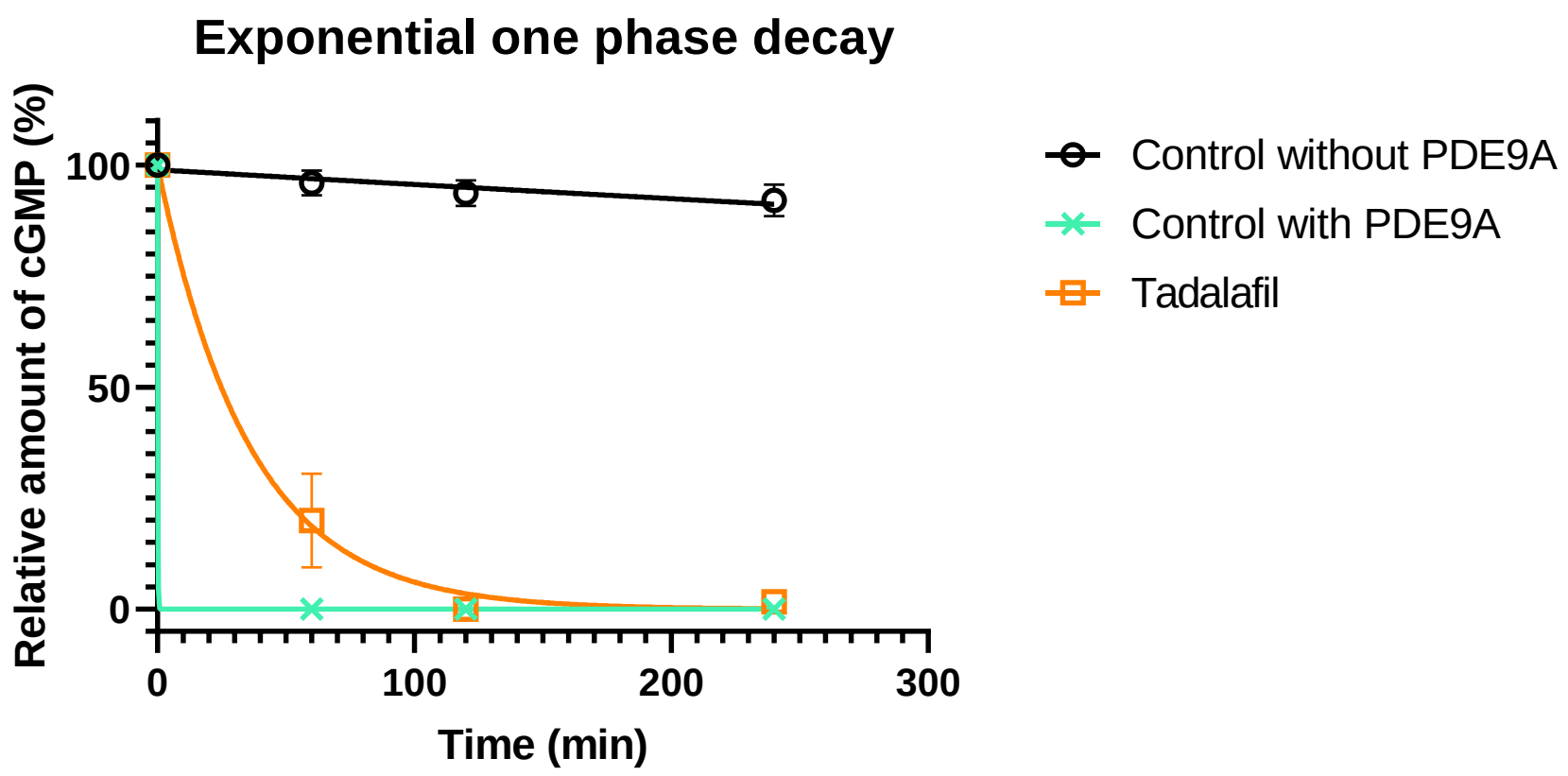

Figure S10. Exponential one-phase decay of cGMP relative concentration over time in the presence of different inhibitors. Curves represent the mean of three independent experiments ± SD. Solid lines correspond to nonlinear regression fits to a one-phase exponential decay model. The control experiments were performed with cGMP in absence or presence of PDE9A, as shown by the black and green lines, respectively. Tadalafil was used as a negative control and is depicted as the orange line.

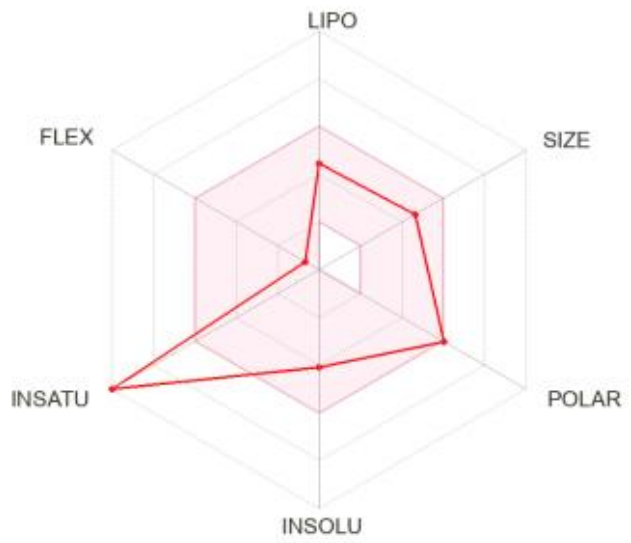

	Molecular weight	H-bond donors	H- bond acceptors	LogP
**560**	381.13 g/mol	5	7	2.07

Figure S11. ADME prediction of compound BS560. On the left, a radar plot showing a summary of ADME properties is depicted. On the right, a table showing the main physico-chemical properties of compound BS560 is reported.
